# Analyses of contiguous reference genomes of *Amaranthus tuberculatus* highlight the landscape of the sex-associated region and PEBP gene family diversity

**DOI:** 10.1186/s12864-025-12181-w

**Published:** 2025-11-03

**Authors:** Damilola A. Raiyemo, Luan Cutti, Eric L. Patterson, Victor Llaca, Kevin Fengler, Jacob S. Montgomery, Sarah Morran, Todd A. Gaines, Patrick J. Tranel

**Affiliations:** 1 Department of Crop Sciences, University of Illinois, Urbana, IL 61801 USA; 2 Department of Plant, Soil and Microbial Sciences, Michigan State University, East Lansing, MI USA; 3 Genome Center of Excellence, Corteva Agriscience, Johnston, IA USA; 4 Department of Agricultural Biology, Colorado State University, Fort Collins, CO USA

**Keywords:** Chromosomal fusion, Dioecy, Retrotransposon, Sex-associated region, Waterhemp genome, Weedy amaranth

## Abstract

**Background:**

*Amaranthus tuberculatus* (waterhemp) is a troublesome agronomic weed species that is dioecious with an XY sex-determination system. The evolution of the sex-determining region (SDR), the contiguity of the region, genomic landscape, and the expression pattern of genes within the region remain poorly understood.

**Results:**

We assembled a high-quality, chromosome-level nuclear genome and chloroplast and mitochondrial genomes of a male *A. tuberculatus*. Combining the genomes with restriction site-associated DNA genome-wide association (RAD-GWA) analysis, comparative genomics, adaptive evolution analysis, and transcriptomic profiling, we identified a ~ 31.8 Mb region on chromosome 1 that is strongly associated with sex. This region is gene-poor, abundant in long terminal repeat (LTR) retrotransposons, and harbors two inversions and a 3.19 Mb haplotype-specific region. Synteny analysis revealed that chromosome 1 likely originated from the fusion of two ancestral chromosomes, and mRNA analysis indicated 76 genes out of the 528 protein-coding genes within the putative SDR of Hap1 were differentially expressed between mature male and female flowers, with several of the genes enriched for Gene Ontology (GO) terms involved in floral development. We further characterized the phosphatidylethanolamine binding protein (PEBP) family in *A. tuberculatus* and related species to gain insights into *FLOWERING LOCUS T* diversity, as well as identified nuclear insertions of organellar origin in the species.

**Conclusion:**

Our results provide insight into the evolution of a sex-associated region in a weedy dioecious species, and the diversity of the PEBP protein family in amaranths. The genomic resources from this study will also be valuable for addressing further questions on adaptive trait evolution within the genus as well as questions surrounding dioecy in this and other plant species.

**Supplementary Information:**

The online version contains supplementary material available at 10.1186/s12864-025-12181-w.

## Background

Dioecy, the separation of male and female reproductive systems on different plants, has evolved multiple times independently across many lineages [1–3], and via several mechanisms [4–7]. One model of dioecy evolution postulates that dioecy could evolve via a gynodioecy pathway requiring two mutations [4, 8, 9] while the second model (one-gene model) postulates that dioecy could evolve via a single regulatory factor [7]. Both models have been supported by the discovery of either two [e.g., in *Actinidia* spp [10]. and *Asparagus officinalis* [11]] or one sex-determining gene(s) [e.g., in *Diospyros lotus* [12] and several *Populus* species [13]].

The sex-determining genes could either be contained within a small region (e.g., ~ 150 kbp in *Vitis* spp.) [14] or a larger non-recombining region that may have different strata (i.e., evolutionary histories) (e.g., 17.42 Mb in *Spinacia oleracea*) [15]. These regions typically accumulate repetitive sequences, possibly due to the low rates of crossing over [16–18]. The study of sex-determining regions (SDR) or the chromosomes containing the regions has been complicated due to assembly challenges posed by gene turnovers, structural variations, and repetitive sequences common within SDRs [19]. Nevertheless, the advances in sequencing technologies and overall improvements in computational approaches have made possible the sequencing and assembly of whole genomes and, more importantly, contiguous sex chromosomes, of non-model species such as weeds [20–24].

*Amaranthus tuberculatus* (Moq.) J.D. Sauer (waterhemp) is a troublesome dioecious weed of agronomic crops, native to the Midwest United States, but now with a global range [25, 26]. It is one of nine dioecious amaranths in the subgenus *Acnida* (L.) [25, 27, 28]. Previous studies investigating sex determination in *A. tuberculatus* confirmed males were the heterogametic sex [29, 30], but the sex chromosome pair were indistinguishable via microscopy [31]. Furthermore, a draft genome of *A. tuberculatus* was assembled into 841 contigs and, together with a *k*-mer-based approach, was used to identify male-specific Y contigs totaling ~ 4.6 Mb in length and containing 147 gene models [32, 33]. One of the male-specific Y contigs contained *FLOWERING LOCUS T* (*FT*), which has been hypothesized to contribute to male fitness or early flowering, given that males flower earlier than females in this species [32]. Analysis of short-read sequencing from three dioecious amaranths, *A. acanthochiton*, *A. cannabinus*, and *A. greggii*, within the same clade as *A. tuberculatus* also revealed the conservation of male-specificity of a copy of *FT* [34].

*FT*-like genes are diverse in their functions with roles mainly in the regulation of flowering time and morphogenesis [35, 36], and are members of the phosphatidylethanolamine binding protein (PEBP) family. The PEBP protein family consists of well-conserved proteins that have been reported across the three domains of life – plants, bacteria and archaea [37, 38]. The PEBP protein family is generally divided into three subfamilies in Arabidopsis: TFL-like, FT-like and MFT-like [39–42]. The TFL-like subfamily comprises three genes, *TERMINAL FLOWER1* (*TFL1*), *ARABIDOPSIS THALIANA CENTRORADIALIS* (*ATC*) AND *BROTHER OF FT AND TFL1* (*BFT*); the FT-like subfamily consists of two genes, *FLOWERING LOCUS T (FT*) and *TWIN SISTER OF FT* (*TSF*); and the MFT-like subfamily contains only one gene, *MOTHER OF FT AND TFL1* (*MFT*). A novel PEBP family member in plants, highly similar to the YY-PEBP (YbhB/YbcL family Raf kinase inhibitor-like protein) in prokaryotes and designated *STEPMOTHER OF FT AND TFL1* (*SMFT*), was recently reported [43]. While several studies have investigated genome-wide PEBP gene family diversity in multiple plant species and reported male-specific copies of *FT*-like genes in *Ficus hispida* [44], *Cannabis sativa* [45] and *Amaranthus* species [32, 34], little is known about the diversity of *FT* genes or the broader PEBP gene family in amaranths.

Sex-determining, or male-specific regions, are characterized by the accumulation of DNA fragments, repeats and pseudogenes, a phenomenon observed in liverwort, *Silene*, *Rumex*, *Coccinea*, and papaya [46–49]. Among these DNA fragments are organelle-derived nuclear insertions, termed NUMTs (nuclear mitochondrial DNA segments) and NUPTs (nuclear plastid DNA segments) [50–52]. The availability of organellar genomes enables the study of nuclear insertions originating from plastids or mitochondria, enhancing the understanding of organelle genome structure while aiding in the decoding of their function, inheritance and evolutionary history [53]. Using a combination of Oxford Nanopore Technologies (ONT) Simplex reads sequencing, Hi-C scaffolding, and Bionano optical mapping, we present haplotype-resolved, chromosome-level nuclear genomes alongside complete chloroplast and mitochondrial genomes of *A. tuberculatus*. This comprehensive dataset facilitates the characterization of the candidate sex-determining region in the genome, including its gene content and repeat structure. It also allows comparison of structural rearrangements between the two haplotype assemblies, provides an in-depth overview of PEBP gene family diversity, and identifies organelle-derived nuclear DNA fragments within the species.

## Results

### Assembly metrics and genome repetitive landscape

**Fig. 1:**
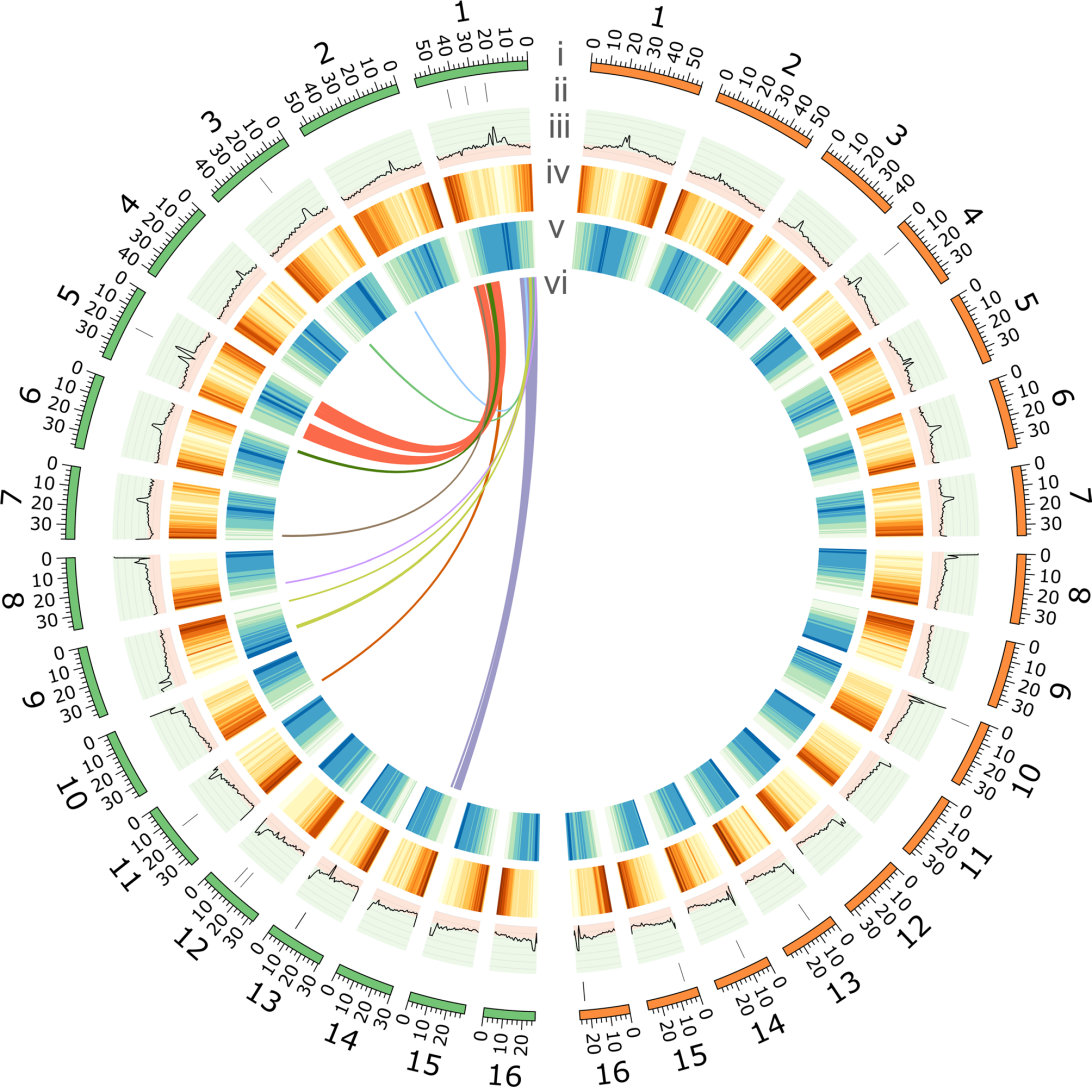
Genomic features of *A. tuberculatus* Hap1 (left) and Hap2 (right) assemblies. Circos plot depicts (i) number and length (Mb) of chromosomes, (ii) distribution of gaps in the genomes, (iii) GC content along the chromosomes, with peaks in light green area representing GC content greater than the median (0.3442) and peaks in light red area representing GC content less than the median, (iv) gene density across the chromosomes, with brown representing gene-rich regions and yellow representing gene-poor regions, (v) LTR (long terminal repeats) density along chromosomes, with blue representing LTR-rich regions and green representing LTR-poor regions, (vi) inner ribbons represent collinear gene blocks on chromosome 1 of Hap1. Collinear genes on chromosome 1 of Hap2 are not shown in the figure to avoid redundancy. Window size of 1 Mb and step size of 500 kbp for ii-iv

Two haplomes (Hap1 and Hap2) consisting of 16 chromosomes each were assembled for a single male individual, representing 90.42% (Hap1) and 86.66% (Hap2) (Table 1) of the estimated genome size of 675.6 Mb from flow cytometry reported by Stetter and Schmid [54]. Analysis of assembly completeness for both Hap1 and Hap2 using BUSCO with embryophyte_odb10 database revealed 97.5% and 97.6% complete BUSCOs, respectively. LTR assembly index (LAI) also revealed high quality assemblies with average LAI scores for Hap1 and Hap2 at 17.98 and 19.28, respectively (Table 1). Repeat analysis using RepeatMasker revealed that 72.01% of Hap1 and 70.88% of Hap2 were made up of repetitive elements. The LTR/*Ty3* elements were the most abundant retrotransposons, representing 13.3% and 11.99% of the Hap1 and Hap2 assemblies, respectively (Table S1). Analysis of high-copy tandem repeats using StainedGlass heatmaps revealed that chromosomes 1–7 appear to be submetacentric, whereas chromosomes 8–16 appear to be telocentric in both haplomes (Fig. S1, Fig. S2). BLAST search of the simple telomeric repeat, TTTAGGG against both haplotypes revealed telomeric repeat sequences at 30 out of the possible 32 telomeric ends for both haplotypes (Table S2), which is comparable to 30 out of 34 telomeric ends reported for *A. tricolor* [55]. Further annotation of the genomes revealed Hap1 and Hap2 had 1,434 and 1,502 genes annotated as transcription factors (TF), respectively (Table S3 and Table S4). Visualization of the analyzed genomic features indicates an inverse relationship between gene density and LTR proportions, whereby gene-rich regions are LTR-poor and LTR-rich regions are gene-poor (Fig. 1).

**Table 1:** Comparison of assembly statistics between the two haplomes of *A. tuberculatus* genome assemblies

Genome characteristics	Hap1	Hap2
Contig assembly size (Mbp)	610.86	585.50
Contig N50 (Mbp)	30.09	35.80
Scaffold assembly size (Mbp)	611.34	585.92
Scaffold N50 (Mbp)	37.57	38.80
Scaffold L50	7	7
GC content (%)	34.94	34.67
Assembly complete BUSCO (%)	97.5	97.6
Size of gaps (Mbp)	0.482	0.420
LTR assembly index (LAI)	17.98	19.28
Annotation characteristics	Hap1	Hap2
Protein-coding genes	28,610	27,824
Mean gene length (bp)	4,808	4,902
Mean CDS length (bp)	1,087	1,094
Mean exon length (bp)	293	292
Mean exon per gene	5.3	5.4
Number of tRNA	2,149	2,071
Total annotated genes	30,759	29,895

### Identification of the candidate sex-determining region

**Fig. 2:**
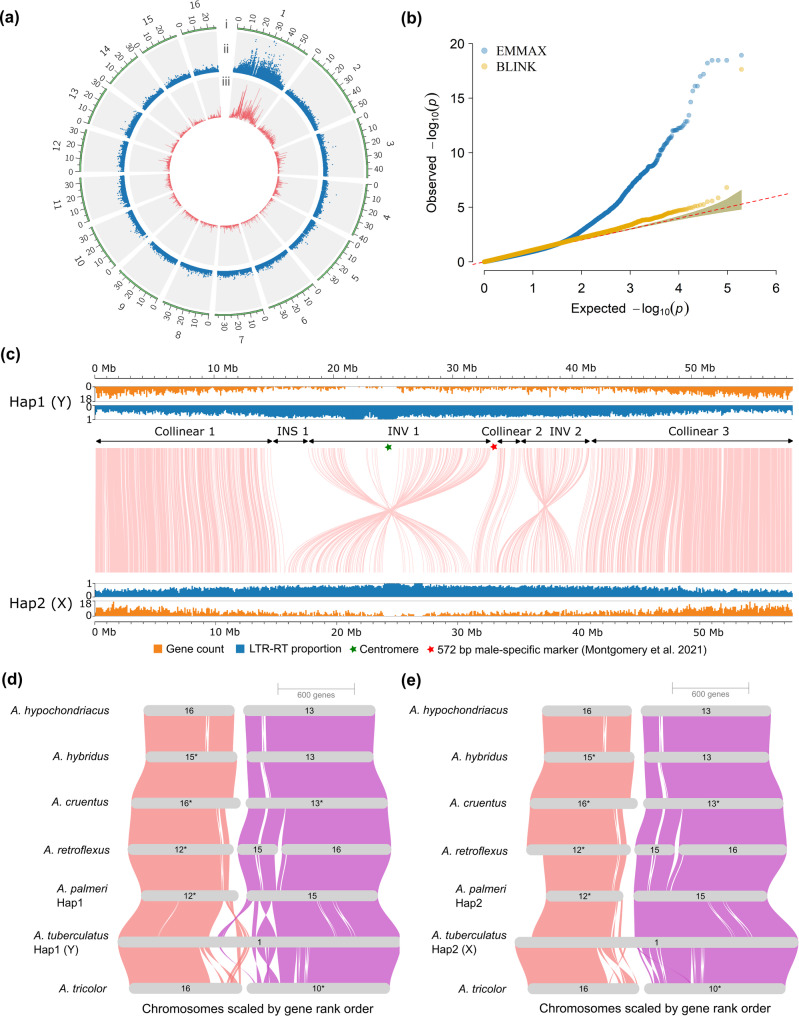
Identification of the sex-associated region on chromosome 1. **a** Circos plot represents (i) number and length (Mb) of chromosomes. (ii) SNPs from GWA analysis using RAD-seq data from 43 females and 45 males. (iii) Fixation index (*F*_*ST*_*)* between females and males across all 16 chromosomes (window size 100 kbp; step size 50 kbp). **b** Quantile-quantile (QQ) plot of two complementary GWA analyses. **c** Synteny plot based on gene order, showing collinear regions, an insertion (INS 1), and two inversions (INV) on chromosome 1. The red asterisk represents a previously reported 572 bp male-specific marker [32] that matched to a region on Hap1. Gene count and LTR-RT proportion were calculated based on 100 kbp non-overlapping windows. **d**, **e** Synteny between the haplotype assemblies of *A. tuberculatus* and chromosome-level assemblies of other *Amaranthus* species highlighting likely fusion of two separate chromosomes in *A. tuberculatus*. Asterisks indicate chromosomes that were manually inverted to keep the gene order consistent with *A. tuberculatus*

Of the 353 individuals genotyped in Montgomery et al. [30], we retained 43 females and 45 males from the population after excluding individuals with more than 60% missing data. Variant calling on these 88 individuals yielded 193,455 variants. Genome-wide association (GWA) analysis with these individuals and variants identified single-nucleotide polymorphisms (SNPs) significantly associated with sex above the Bonferroni threshold (–log_10_*P* = 6.5876). These SNPs were located on chromosome 1, spanning from 14.02 to 45.81 Mb (Fig. 2a). Additional complementary GWA analysis using BLINK revealed a single SNP at position 16,139,826 on chromosome 1 as significantly associated with sex. However, the SNP resides within an intergenic region. The SNP was also the 7th most significant SNP from the first analysis with EMMAX. QQ plots comparing the two GWA approaches revealed underlying population structure, which was accounted for by the different models (Fig. 2b). Genetic differentiation (*F*_*ST*_) along chromosome 1 between females and males exceeding the top 5% threshold spanned from 15.1 to 44.6 Mb, with a second peak at 52.6–52.7 Mb (Fig. 2a).

A BLAST query of previously reported primer sets, used to amplify male-specific regions [32], against both haplotypes also revealed perfect matches to chromosome 1 on Hap1, but none on Hap2. One set of primers (WHMS) matched to a 572 bp region (33,273,720–33,274,292 bp) (Fig. 2c) while primer sets MU-976, MU-657.2, and MU-533 also matched to regions within the candidate SDR on Hap1 (Table S5). The 572 bp male-specific marker had homology to an LTR/*Ty3* retrotransposon (Fig. S3). Similarly, MU-657.2 was previously reported to have homology to a LTR/*Ty3* element in sugar beet [30], indicating the abundance of the *Ty3* superfamily within the SDR. A BLAST search of a 200 bp *FT* gene, reported to be male-specific across three dioecious amaranths related to *A. tuberculatus* [34], against both haplotype assemblies also revealed a perfect match to the fourth exon (15,545,318–15,545,518 bp) of a full-length, four-exon gene, AmaTuChr01Ag009380 on Hap1. We therefore consider chromosome 1 of Hap1 the Y and chromosome 1 of Hap2 the X (Fig. 2b).

Reciprocal best hit (RBH) search between 147 genes from previously identified male-specific contigs in a draft *A. tuberculatus* genome [32] and the haplotype assemblies from this study revealed 18 and 12 genes had best matches on chromosome 1 of Hap1 and Hap2 assemblies, respectively (Table S6). The genes are thus conserved between the draft assembly and the new assembly. Taken together, the line of evidence above supports chromosome 1 as the likely sex chromosome, and we defined an approximate boundary of the candidate SDR as a region of chromosome 1 on Hap1 between 14.02 and 45.81 Mb (~ 31.79 Mb). There are 2,614 protein-coding genes on chromosome 1 of Hap1, of which 796 are within the candidate SDR.

### Comparative analysis between the candidate SDR of the two haplomes

Synteny patterns between the two haplomes indicate a 1:1 relationship in gene content (Fig. S4). Out of the 20,763 gene pairs representing the reciprocal best matches between the two haplotypes, there were 1,822 pairs for chromosome 1, which were used to define the boundaries of regions within the candidate SDR (Collinear 1: 663, INV 1: 131, Collinear 2: 21, INV 2: 94, Collinear 3: 913). The synteny analysis revealed two inversions on either side of a collinear region (Collinear 2) within the candidate SDR (Fig. 2c). One inversion (INV 1) is from 17,907,092–33,155,961 bp on Hap1, and from 15,719,009–31,029,795 bp on Hap2, based on the first and last gene pairs for the inversion. The other inversion (INV 2) ranges from 35,809,637–41,407,537 bp on Hap1, and from 33,229,536–39,616,194 bp on Hap2 (Fig. 2c). Further analysis of structural rearrangements using SyRI revealed 274 inversions spread across the 16 chromosomes between Hap1 and Hap2 (Table S7); however, INV 1 and INV 2 within the SDR were the two largest inversions out of the 274 inversions identified using SyRI (Table S7). Comparison of chromosome 1 across *A. tuberculatus* and six other *Amaranthus* species indicates a conserved gene order between the species and Hap2; however, it appears the inversions likely occurred on Hap1 (Fig. 2d and e). Also, chromosome 1 appears to have originated from the fusion of two ancestral chromosomes (Fig. 2d and e).

**Fig. 3:**
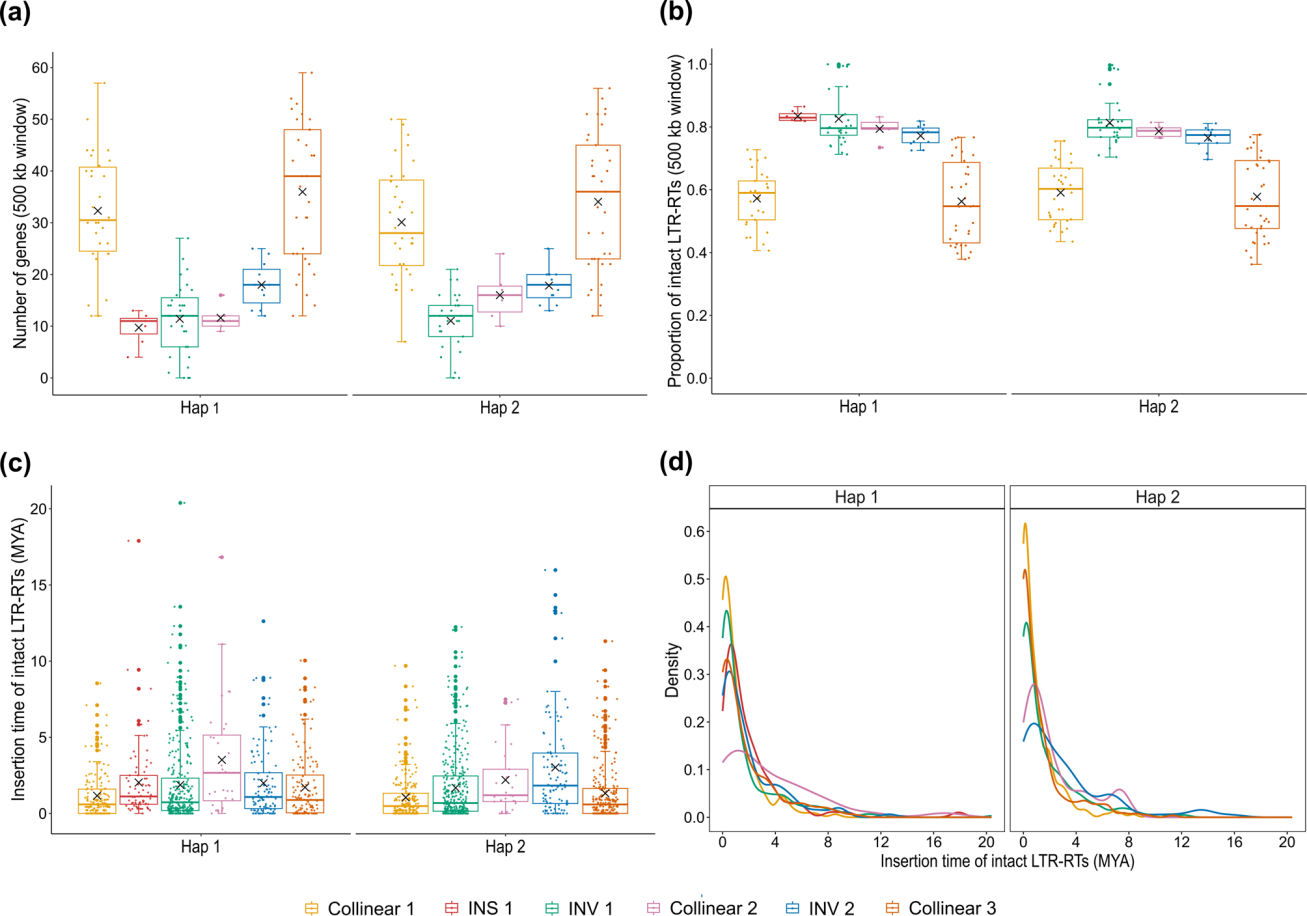
Comparative analysis between chromosome 1 of the two haplomes. **a** Number of genes across five regions (three collinear regions and two inversions) plus an insertion in Hap 1 on the chromosome. Gene densities are calculated per 500 kbp non-overlapping windows. **b** Proportion of intact LTR-RTs across the five regions on the chromosome. LTR-RT proportions are calculated per 500 kbp non-overlapping windows. **c** Insertion time of intact LTR-RTs across the five regions. **d** Density distribution of intact LTR-RTs insertion times across the five or six regions on the chromosome

Analysis of the inversions revealed INV 1 on Hap1 and Hap2 contain 326 and 313 protein-coding genes, respectively, although only 130 genes are syntenic between the haplotypes. INV 2 on Hap1 and Hap2 contain 191 and 215 protein-coding genes, respectively, with only 93 syntenic genes between the haplotypes. A region upstream of Hap1 between 14,660,708–17,851,789 bp (3.19 Mb) was found to lack synteny to any region of chromosome 1 on Hap2, and no homologous collinear block of sequences elsewhere in the genome. This region was designated INS 1 and contains 58 protein-coding genes (Fig. 2e; Table S8). However, only 5 of the genes (encoding EG45-like domain containing protein 1, FT/HEADING DATE 3 A, FLOURY 1, polyadenylate-binding protein 5, and binding partner of ACD11 1) were annotated while the rest were uncharacterized or unknown. Additional analysis of the 58 genes within INS 1 revealed 35 genes had homologous copies across the 16 chromosomes with greater than 70% similarity at the protein sequence level (Table S9). Although the *FT/HEADING DATE 3A* and its homologs vary in their intron length, they appear to have a conserved gene structure (Fig. S5). Phylogenetic analysis of the encoded protein sequences of the *FT/HEADING DATE 3A* and its homologs indicate that the copy (AmaTuChr01Ag009380) within the male-specific Y region (MSY) of *A. tuberculatus* Hap1 genome is more related to the copy (AmaPaChr00Ag260700) in unplaced chromosome (Chr00) of the *A. palmeri* genome rather than the copy (AmaPaChr03Ag064170) within the MSY of *A. palmeri* (Fig. S5). Gene density for the regions (collinear 1, INS 1, INV 1, collinear 2, INV 2, and collinear 3) on chromosome 1 of Hap1 and Hap2 were statistically different with *p* < 6.056e-14 and *p* < 1.133e-13, respectively (Fig. 3a). Similarly, intact LTR proportions for the regions were statistically different for both haplotypes (Hap1; *p* < 2.341e-16 and Hap2; *p* < 2.85e-15) (Fig. 3b). Pairwise comparison indicated that collinear 1 and 3 are gene-rich but LTR-poor while the regions (INS 1, INV 1, collinear 2, and INV 2) are LTR-rich but gene-poor for Hap1, which was same for Hap2 (excluding the insertion which was not present in the haplotype). Although analysis of insertion times revealed slight variation in the timing of intact long terminal repeat retrotransposons (LTR-RTs) insertions for collinear 2 and INV 2 compared with the other regions, both haplotypes appear to have accumulated several intact LTR-RTs in these regions within the last 0.5 MYA, as indicated by the peaks around this time (Fig. 3c and d).

### Sequence divergence and detection of genes under positive selection

Analysis of adaptive evolution using the M1a vs. M2a model revealed 24 genes out of 608 single-copy genes on chromosome 1 were significant (α = 0.05) for sites under positive selection (Table S10). Using the branch model, where Hap1 was used as the foreground branch to determine if ω differs for this branch relative to the background branches (i.e., the M0 vs. two-ratio model), 5 genes, including a serine/arginine-rich SC35-like splicing factor 33 (*SCL33*) had significantly different ω, indicating it confers some fitness benefit. When Hap2 was used as the foreground branch, only three genes had significantly different ω. When both haplomes were specified as the foreground branch [i.e., (Hap1 #1, Hap2 #1)], eight genes had ω that differed for the specified branch. Similarly, when the branch leading to the common ancestor of the two haplomes were included in the foreground branch (i.e., (Hap1 #1, Hap2 #1) #1)], eight genes, including ones encoding bark storage protein B and polygalacturonase, had ω that differed for the foreground branch compared to the background branches.

Adopting the foreground and background branches used for the branch model above but utilizing a branch-site model (i.e., MA_null_, ω = 1 vs. MA, ω > 1), indicated 24 genes, 35 genes, 50 genes, and 46 genes were more likely to contain sites with ω > 1 when Hap1, Hap2, both haplotypes, the branch leading to the common ancestor, and the branch leading to the haplomes was used as the foreground branch, respectively (Fig. S5, Table S10, and Table S11). Within the candidate SDR, genes encoding E3 SUMO-protein ligase SIZ1-like, arabinogalactan peptide 16, and several unknown proteins were more likely to contain sites under positive selection based on the branch-site model if Hap1 was the foreground branch (Fig. S6, Table S10, and Table S11). Taken together, our analysis above reveals genes that are potentially important in sex-specific adaptation.

### Expression analysis identifies genes involved in floral development

**Fig. 4:**
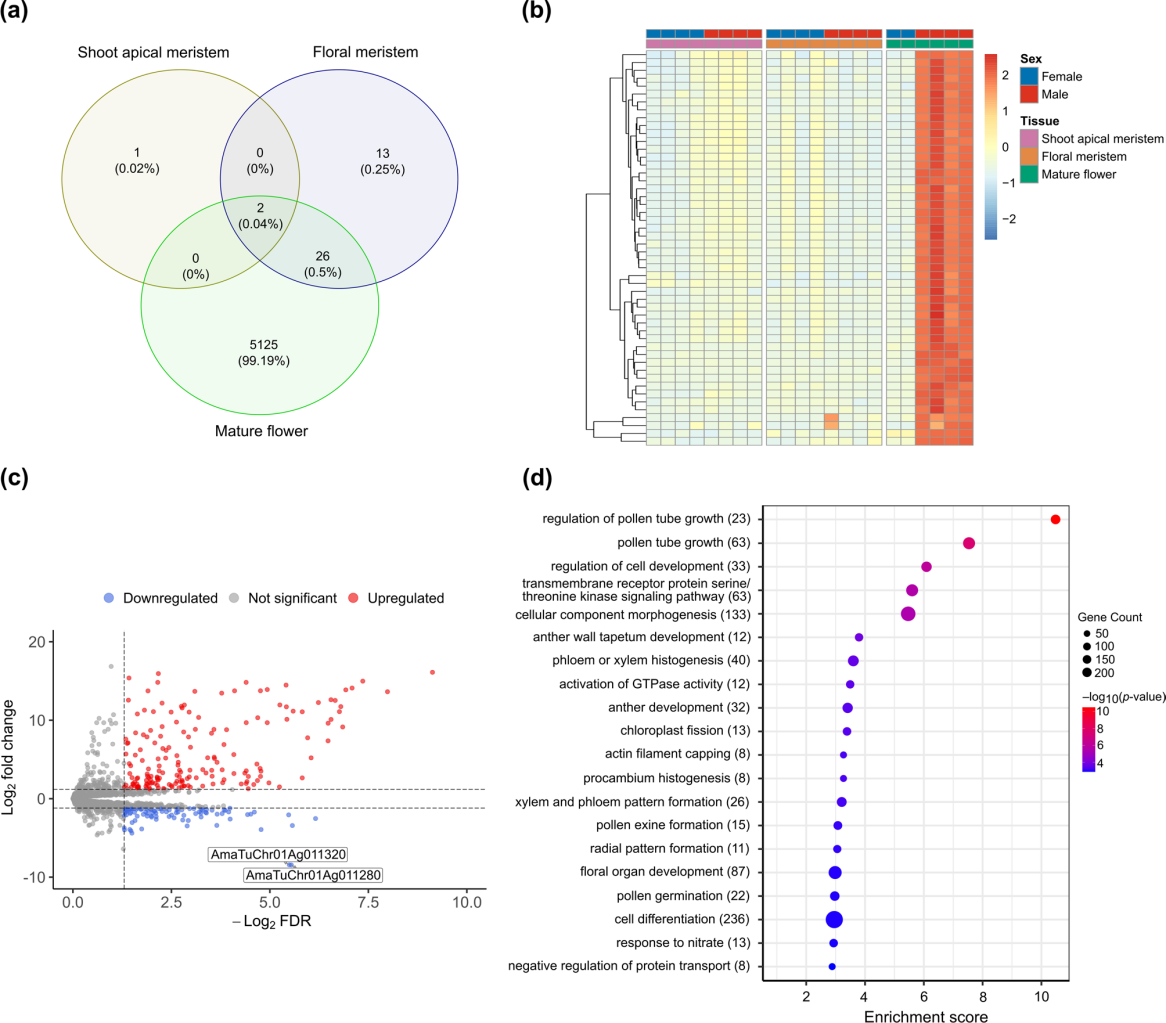
Differential gene expression analysis between male and female individuals across three tissue types. **a** Numbers of differentially expressed genes for male versus female comparison for shoot apical meristem, floral meristem, and mature flower. **b** Heatmap of rlog (regularized log) transformed raw counts of the 5,153 differentially expressed genes in male versus female comparison for mature flower. Samples are ordered using the hierarchical clustering method. Brown depicts genes with relatively high expression, yellow depicts intermediate expression levels and blue represents genes with relatively low expression levels. **c** Volcano plot of differentially expressed genes on chromosome 1 for mature flower. Highlighted genes were consistently downregulated among the three tissue types highlighted. Dotted lines represent FDR < 0.05 and FC > 1.2 thresholds. **d** Gene ontology enrichment analysis showing significantly overrepresented terms for male versus female differentially expressed genes for mature flower. Values in parentheses represent the number of genes within the term

Cleaned mRNA reads (i.e., adapter-trimmed and low-quality bases removed) from three tissue types (shoot apical meristem, floral meristem, and mature flower) reported by Bobadilla et al., [56] that were mapped to the Hap1 assembly had uniquely mapped reads for males ranging from 84.01 to 89.45%, and uniquely mapped reads for females ranging from 74.82 to 90.54%. Out of the 28,610 annotated Hap1 protein-coding genes, 21,530 genes were retained for differential expression analysis after filtering and TMM normalization (Fig. 4a). Among the retained genes, 5,153 were differentially expressed between male and female individuals at the mature flower stage, representing the majority of differentially expressed genes across all sampled tissue stages in the study (Fig. 4b). Among the 1,904 genes retained on chromosome 1, two at the shoot apical meristem stage, four at the floral meristem stage, and 446 (244 upregulated and 202 downregulated) at the mature flower stage were differentially expressed between males and females (Fig. 4c; Table S12 – S14). Within the candidate SDR on chromosome 1, two genes at the shoot apical meristem stage, two genes at the floral meristem stage, and 76 genes (44 upregulated and 32 downregulated) at the mature flower stage were differentially expressed (Table S12 – S14). Two genes within the candidate SDR (specifically within INV 1) encoding MADS-box transcription factor 23 (AmaTuChr01Ag011280) and LOB domain-containing protein 19-like (AmaTuChr01Ag011320) were consistently downregulated across the three tissue types for male plants (Table S12 – S14). Gene ontology (GO) term enrichment analysis was performed to gain insight into biological processes that could be involved in sex determination. Biological processes including pollen tube growth, pollen germination, pollen exine formation, regulation of cell development, cellular component morphogenesis, and anther wall tapetum development were identified among the top 20 enriched GO terms (Fig. 4d, Table S15 – S16). The top five terms enriched for molecular function included transmembrane receptor protein serine/threonine kinase activity, GTPase activator activity, small GTPase binding, pectinesterase activity and mannan synthase activity (Table S17), while the top five terms enriched for cellular function included plasma membrane, apical plasma membrane, pollen tube, pollen tube tip, and transport vesicle (Table S18).

### PEBP gene family diversity

**Fig. 5:**
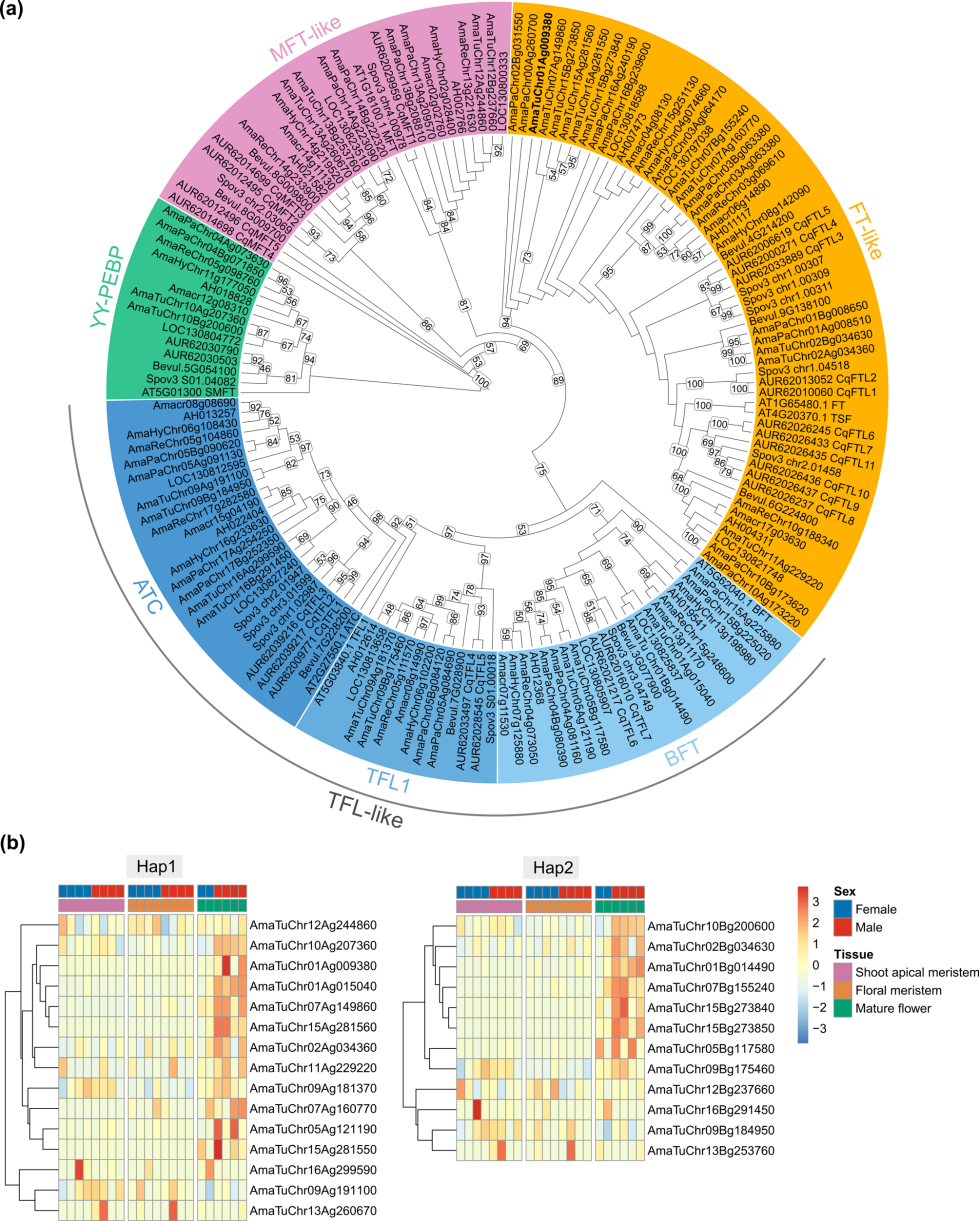
PEBP protein family diversity and corresponding expression patterns in *A. tuberculatus*. **a** Phylogenetic tree of PEBP proteins from *Amaranthus* species, *Chenopodium quinoa*, *Spinacia oleracea*, *Beta vulgaris* and *Arabidopsis thaliana*. Numbers above branches represent RAxML bootstrap support (BS) values. Only BS greater than 45% are shown on tree. Subfamilies are indicated as TFL-like (TERMINAL FLOWER1-like; includes *ATC* [*ARABIDOPSIS THALIANA CENTRORADIALIS*], *TFL1* [*TERMINAL FLOWER1*], and *BFT* [*BROTHER OF FT AND TFL1*]), MFT-like (MOTHER OF FT AND TFL1-like), FT-like (FLOWERING LOCUS T-like), and YY-PEBP (YbhB/YbcL family Raf kinase inhibitor-like protein). The male-specific FT-like copy on Hap1 is shown in bold. **b** Heatmap indicating expression patterns of *PEBP* genes in *A. tuberculatus* Hap1 and Hap2 across three tissue types

The identification of a male-specific *FT* copy in *A. tuberculatus*, along with the conservation of the *FT* in three related species and the hypothesis of its evolutionary benefits in male fitness, highlights the need for a comprehensive exploratory analysis of *FT* genes and the PEBP gene family in amaranths. BLAST and HMMsearch analyses of PEBP genes in the amaranths revealed several candidates; however, only genes with PEBP conserved domains were retained for downstream analysis. We identified 9 (*B. vulgaris*), 10 (*A. hybridus*), 11 (*A. hypochondriacus*, *A. cruentus*, *A. retroflexus*, and *A. tricolor*), 12 (*A. tuberculatus* Hap2), 13 (*A. palmeri* Hap2), 14 (*A. palmeri* Hap2), and 15 (*A. tuberculatus* Hap1) PEBP genes across the species. The genes were further classified into subfamilies based on the Arabidopsis-derived classification system, namely MFT-like, TFL-like, and FT-like (Fig. 5a). The *MFT* gene had two copies in *Amaranthus* species, *B. vulgaris* and *S. oleracea*, compared with a single copy in *A. thaliana* and five copies in *C. quinoa*. Our analysis also distinguished clearly between *TFL1*, *ATC*, and *BFT* genes (members of the TFL-like subfamily) across the species (Fig. 5a). All the species had a single copy of *TFL1*, compared to two copies in *C. quinoa*. The *Amaranthus* species had two copies, each of *ATC* and *BFT*, while *B. vulgaris* had a single copy of each. The FT-like subfamily was more diverse than the other two subfamilies. There was also no clear distinction between the *FT* and *TSF* genes (both members of the FT-like subfamily). Our analysis revealed two copies of FT-like genes in *A. hybridus*; three copies in *A. hypochondriacus*, *A. cruentus*, *A. tricolor*, and *A. retroflexus*; four copies in *B. vulgaris*; five copies in *A. palmeri* Hap2; and six copies in *A. palmeri* Hap1, compared with five copies in *S. oleracea* and eleven copies in *C. quinoa*. In Hap1 of *A. tuberculatus*, there were seven copies of FT-like genes while Hap2 had four copies of the FT-like genes. Both AmaTuChr01Ag009380 and AmaTuChr01Ag015040 are within the candidate SDR on chromosome 1 of Hap1; however, only AmaTuChr01Ag009380 was Hap1-specific and previously reported to be male-specific while AmaTuChr01Ag015040 is homologous to AmaTuChr01Bg014490 in Hap2. Among the PEBP genes in *A. tuberculatus*, only two (AmaTuChr10Ag207360 in Hap1 and AmaTuChr10Bg200600 in Hap2; annotated as uncharacterized protein, and AmaTuChr01Ag015040 in Hap1 and AmaTuChr01Bg014490 in Hap2; annotated as CEN-like protein 1) were upregulated in mature male flowers relative to female flowers (Fig. 5b). Based on the NCBI Conserved Domain Database (CDD), the uncharacterized protein contains a domain homologous to the PEBP domain found in bacteria and archaea, and is classified as a YbhB/YbcL family Raf kinase inhibitor-like protein, similar to *E. coli* YbhB and YbcL. Genes from other amaranths clustering with AmaTuChr10Ag207360 also contain a domain homologous to a PEBP domain in bacteria and archaea; we therefore designate this clade YY-PEBP in our phylogenetic tree (Fig. 5a).

### Organellar genome features and their homologous sequences in the nuclear genome

**Fig. 6:**
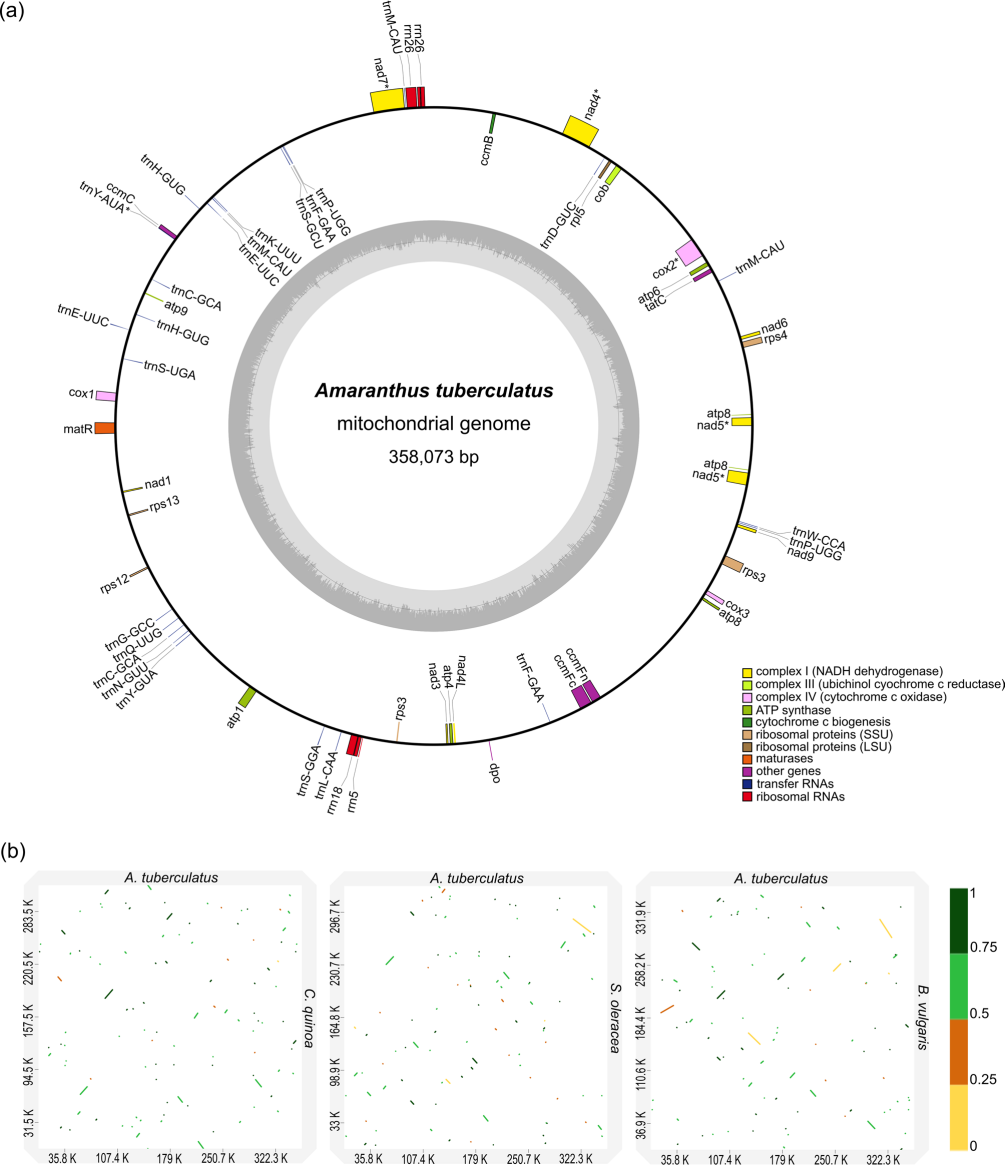
Mitochondrial genome of *Amaranthus tuberculatus* and comparisons with related species. **a** Circular structure of the *A. tuberculatus* mitochondrial genome. Genes shown on the outside of the map are transcribed clockwise while genes shown on the inside are transcribed counterclockwise. The dark grey region within the circle represents GC content. **b** Dotplot visualization of alignment of the *A. tuberculatus* mitochondrial genome to related species. The scale bar represents percentage identity

Due to several recent reports of chloroplast genomes for *A. tuberculatus* [57, 58], we focused on mitochondrial genome features of the species but further explored insertions from both organelles into the nuclear genome, including within the candidate SDR. The mitochondrial genome of *A. tuberculatus* was assembled to a length of 358,073 bp, and contained 29 protein-coding, 25 transfer RNA (tRNA) and 3 ribosomal RNA (rRNA) genes (Fig. 6a; Table S19). Sixty-four simple sequence repeats were identified in the mitochondrial genome, in which tetranucleotide (37) were most abundant, followed by trinucleotide (11), mononucleotide (9), dinucleotide and pentanucleotide (3), and hexanucleotide (1). Additional repeat analysis using REPuter revealed the mitochondrial genome had 31 forward repeat sequences and 69 palindromic repeat sequences. Comparison of the *A. tuberculatus* mitochondrial genomes to mitochondrial genomes from *C. quinoa*, *S. oleracea*, and *B. vulgaris* indicates a high degree of rearrangements and low conservation among the species (Fig. 6b).

Analyses of mitochondrial DNA insertions revealed 14 mitochondrial genes (*atp1*, *atp8*, *ccmFC*, *ccmFn*, *cob*, *cox1*, *cox3*, *nad3*, *nad4*, *nad5*, *nad9*, *rpl5*, *rps3*, *rps4*) had copies distributed across 9 chromosomes in Hap1 (Table S20A). Similarly, 14 mitochondrial genes (*atp1*, *atp8*, *ccmC*, *ccmFC*, *ccmFn*, *cox1*, *nad2*, *nad4*, *nad5*, *nad9*, *rpl5*, *rps12*, *rps3*, *tatC*) had copies distributed across 12 chromosomes in Hap2 (Table S20B). Analyses of chloroplast DNA insertions revealed Hap1 and Hap2 had 59 and 61 chloroplast genes, respectively, distributed across the 16 chromosomes (Tables S21 and S22). Among the chloroplast genes integrated into the nuclear genomes, *ycf2*, *ycf1*, and *rpoC2* were most abundant with greater than 20 copies (Tables S21 and S22). A search of the chloroplast genes within the mitochondrial genome revealed 24 genes (*accD*, *atpB*, *atpE*, *atpF*, *atpH*, *atpI*, *cemA*, *ndhB*, *pafII*, *petA*, *petG*, *psaI*, *psbA*, *psbC*, *psbE*, *psbF*, *psbL*, *rbcL*, *rpl2*, *rpoC1*, *rpoC2*, *rps2*, *ycf1*, and *ycf2*) were found within the mitochondrial genome; however, none of the mitochondrial genes had a sequence match in the chloroplast genome (Table S23). Analysis of larger blocks of DNA fragments revealed 29 fragments with a total length of 33.38 kbp from the chloroplast genome had homologs in the mitochondrial genome (9.32% of the mitochondrial genome) (Table S24). Several fragments (> 400) from both the mitochondrial and chloroplast genomes were found to have integrated into the nuclear genome with longer stretches in some cases (e.g., an 18.25 kbp chloroplast fragment integrated into chromosome 2 of Hap1, an 11.14 kbp chloroplast fragment integrated into chromosome 6 of Hap2, a 9 kbp mitochondrial fragment integrated into chromosome 3 of Hap1, and a 42.56 kbp mitochondrial fragment integrated into chromosome 13 of Hap2) (Supplementary Table S25 – S28). Within the candidate SDR (14.02–45.81 Mbp) in Hap1, about 5,058 bp of mitochondrial sequences (including *atp1*, *nad3*, and *rps3* gene fragments totaling 1,150 bp) were inserted into the region. About 3,188 bp of chloroplast sequences (including *atpI*, *ndhB*, *ndhH*, *psbC*, and *ycf2* totaling 1,038 bp) were also inserted into the SDR on Hap1 (Supplementary Table S25 – S28). No mitochondrial or chloroplast sequence fragments however had a match to any sequence within the region (14.66–17.85 Mb) designated as male-specific (INS 1) on Hap1. Similarly, within the region on Hap2 (~ 14.6–43.00 Mb) equivalent to the candidate SDR on Hap1, about 6,731 bp of mitochondrial sequences (including the *rps3* gene fragment totaling 816 bp) were inserted into the region. Also, about 7,383 bp of chloroplast sequences (including *accD*, *matK*, *ndhF*, *psaB*, *ycf1*, and *ycf2* totaling 5,526 bp) were inserted into the region (Supplementary Table S25 – S28).

## Discussion

We present a haplotype-resolved nuclear genome and chloroplast and mitochondrial genome assemblies of *A. tuberculatus* with high contiguity. We comprehensively demonstrate that chromosome 1 of the assembled genome contains the candidate region that is strongly associated with sex, harboring the two largest inversions in the genome within a ~ 31.8 Mb region that is gene-poor but abundant in LTR retrotransposons. The regions flanking this candidate sex-determining region are gene-rich and LTR-poor, indicating the possibility that they are still actively recombining between the Y and the X. This observed SDR landscape is also corroborated by evidence from *Spinacea oleracea*, where two inversions within a 17.42 Mb are flanked by pseudoautosomal regions (PAR) that are syntenic between the Y and X [15]. The concentration of high-order repeats, typical of centromeric regions [59], in the middle of chromosome 1 indicates the chromosome is either submetacentric or metacentric, with the first inversion (INV 1) located in the peri- or centromeric region of the chromosome. Inversions have been observed within sex chromosomes of different species, including plants and animals [15, 47, 60, 61], and are known evolutionary drivers of sex determination systems [60]. Chromosome 1 also appears to have originated from the fusion of two ancestral chromosomes following the divergence of the *Acnidia* subgenus from *Albersia*. This fusion (referred to as Robertsonian translocation) was independently observed by Kreiner et al. [62] in their study of sex evolution in *A. tuberculatus*, in which they also identified chromosome 1 as the sex chromosome. In that study, the authors report a smaller (~ 3 Mb) region on the Y chromosome that is strongly but incompletely associated with maleness. While Robertsonian translocation typically involves the fusion of two telocentric or acrocentric chromosomes [63], we note that in *A. tricolor*, chromosome 10 appears to be telocentric [55], and therefore suitable for fusion; however, chromosome 16 appears to be metacentric or submetacentric [55], and initial shortening of the chromosome arm or a complex mechanism would be required for the fusion of both chromosomes. Interestingly, chromosome 1 of *A. tuberculatus* shows two regions with high-order repeats and thus appears dicentric; however, whether both centromeres are active, functional or stable would require further investigation. It is worth noting that Kreiner et al. [62] reported varying SDR architectures among several populations, with an inversion segregating between X and Y across three haplotypes and thus reflects population-level diversity of the SDR in *A. tuberculatus*.

In multiple species, insertions, notably TE accumulation, have contributed to the expansion of sex-determining regions [16, 64–66]. Although the haplotype-specific insertion (INS 1) upstream of the first inversion has accumulated retrotransposons, the region still contained fifty-eight genes, including *FT*/*HEADING DATE 3A*, which we previously reported as male-specific in this species [34]. Given that 50% of the genes have homologous copies elsewhere in the genome, and none of the genes are expressed across three tissue types, it is possible that the genes are not functional or perhaps their expression was not captured at the stage during which tissues were sampled. Furthermore, only two genes within the sex-determining region on chromosome 1, one encoding a MADS-box transcription factor 23 and the other, a LOB domain-containing protein 19-like were downregulated in males across the three tissue types. Further comparison between this work and Bobadilla et al. [56] who investigated the expression of genes between male and female individuals using a draft *A. tuberculatus* contig assembly indicates high congruency in the expression patterns observed in both studies. While the authors reported no differentially expressed genes within the putative male-specific Y contigs from the draft assembly, they found autosomal genes, including MADS-box transcription factor 18 (*MADS18*), LOB domain-containing protein 31 (*LOB31*), *SOC1*, and *JAGGED* that were downregulated in males. These genes were also downregulated in our reanalysis of their transcriptome data; however, the genes are now mapped to their respective chromosomal locations. Similar to their finding, several genes, including *PMADS2*, *CMB2*, *bHLH91*, and *CYP120A11*, reported as upregulated in males were also upregulated in males in this study. It has been suggested that the transition to dioecy in *A. tuberculatus* is incomplete and ongoing, with the Y and X chromosomes still in an early stage of divergence (proto-Y/X) [62]. Consistent with this, our finding that intact LTR retrotransposon accumulation dated to less than 0.5 MYA within the candidate region associated with sex supports the hypothesis that recombination suppression within this region is relatively recent. Waselkov et al. [67] estimated the stem age of *Amaranthus* at 16.2–11.1 MYA, while Wang et al. [55] estimated that *A. tuberculatus* diverged from *A. palmeri* and monoecious amaranths 5.29 MYA. It therefore appears that most of the intact LTR insertions into the sex-associated region occurred after speciation. Nevertheless, the conservation of sex markers, including *FT*, among three species (*A. acanthochiton*, *A. cannabinus*, and *A. greggii*) closely related to *A. tuberculatus* [68] suggests that the sex-associated region is more ancient, potentially preceding the divergence of these species.

While numerous studies have demonstrated the significance of PEBP proteins in the growth and development of multiple plant species, few studies have investigated the diversity of the PEBP protein family in *Amaranthus* species. We leveraged the high-quality chromosome-level genomes of species within the genus to bridge this knowledge gap. Our genome-wide PEBP protein analysis suggests several factors (e.g., lineage-specific duplications, retention bias following whole genome duplication, or transposon-mediated duplications) could have given rise to the additional copies of *PEBP* genes (specifically *FT*-like) in *A. tuberculatus* and *A. palmeri*, compared to the other *Amaranthus* species. The imbalance in *FT*-like gene copies between Hap1 and Hap2 of *A. tuberculatus* may however be due to either haplotypic differences or fragmentation of the Hap2 assembly. Despite significant advances in genome sequencing and assembly, contiguous assembly and accurate phasing are still major challenges for complex genomes [69, 70]. Although only two genes; a *BFT* gene (AmaTuChr01Ag015040 in Hap1 or AmaTuChr01Bg014490 in Hap2) and a *YY-PEBP* gene (AmaTuChr10Ag207360 in Hap1 or AmaTuChr10Bg200600 in Hap2), among the *PEBP* genes were upregulated in mature male flowers, it is possible that the *FT*-like genes have time-specific or tissues-specific expression patterns not detected in our mRNA data. While *BFT* promotes meristem growth in plants [71], *YY-PEBP* (designated *STEPMOTHER OF FT AND TFL1*) represents a novel member of the PEBP gene family that appear to have originated via horizontal gene transfer (HGT) from a prokaryote into an ancestral streptophyte, although its evolutionary origin within Viridiplantae remains unclear [43]. Further analyses suggest that this *YY-PEBP* gene may contribute to the maintenance of reproductive structure vitality and is present as a single copy [43]. Although the function of *YY-PEBP* in amaranths has not yet been determined, our results indicate that it also exists as a single copy in this lineage. Additional analysis of the FT protein sequence in the amaranths revealed that the copy within the MSY of *A. tuberculatus* is closely related to a copy located on Chr00 (unplaced contigs) in *A. palmeri*, with 67% bootstrap support. These two copies likely share a common ancestor but have since diverged. It is thus possible that these *FT* gene copies confer some male fitness advantage by promoting earlier flowering of males in both species, as previously hypothesized [32, 34]. Overexpression of *FT1* has been shown to induce early flowering in several species e.g., *Manihot esculenta* [72], and *Perilla frutescens* [73]. In *Beta vulgaris*, *BvFT1* (Bevul.9G138100) represses flowering and contributes to vernalization, whereas *BvFT2* (Bevul.4G214200) promotes flowering [74]. *BvFT1* is orthologous to *CqFTL1/CqFTL2* in *C. quinoa* [75] and to *CrFTL2* in *C. rubrum* [76], while *BvFT2* is orthologous to *CqFTL4/CqFTL5* or *CrFTL1* [75, 76]. Notably, *CqFTL1/CqFTL2* are also referred to in the literature as *CqFT1A/CqFT1B-1/CqFT1B-2*, and *CqFTL4/CqFTL5* as *CqFT2A/CqFT2B* [77]. Our analysis revealed that *CqFTL1/CqFTL2* and *CqFTL4/CqFTL5* have homologous copies in the amaranths; however future work incorporating codon-based alignments may capture more recent substitutions and thereby improve the resolution of phylogenetic relationships among taxa.

Understanding how mitochondrial or chloroplast DNA segments integrate into the nuclear genome can reveal the mechanisms of genetic transfers, how gene functions are regulated, or provide insights into genome evolution and adaptation [78–81]. Numerous studies have revealed DNA insertions of organellar origin into the nuclear genome, including sex chromosomes or sex-determining regions, in multiple species [82–84]. The mitochondrial and chloroplast genomes of *A. tuberculatus* (~ 360 kbp) assembled in this study allowed for investigation of these nuclear integrants originating from the organelles. While the mitochondrial genome is comparable in length to other species within the Amaranthaceae family (*B. vulgaris* ssp. *vulgaris*; 368.8 kbp, *B. vulgaris* ssp. *maritima*; 364.96 kbp, *S. oleracea*; 329.6 kbp, and *C. quinoa*; 315 kbp) [85–87], there were differences in the number of protein-coding genes, specifically, *rps7* was not annotated in the *A. tuberculatus* mitogenome, or detected via a BLAST search of the *Chenopodium quinoa rps7* copy to the mitogenome. It is possible that the *rps7* gene is lost in *A. tuberculatus*; however, additional mitochondrial genomes of species within the genus would be required to ascertain if the absence of the gene is lineage specific. Like *B. vulgaris* and *C. quinoa*, the *A. tuberculatus* mitochondrial genome also contains two tRNA^Cys^ genes (*trnC-GCA*): one around 151.7 kbp and the other at 217.3 kbp, which had only been reported in *B. vulgaris* [86] and *C. quinoa* [85]. Although mitochondrion-to-plastid DNA transfer has been documented for some lineages [88], our analysis revealed a unidirectional migration of DNA segments from chloroplast to the mitochondrial genome, as reported for multiple plant species [88–91]. Overall, < 0.02% of the SDR on Hap1 is made up of NUMTs, while 0.01% of the region is made up of NUPTs. The absence of both NUMTs and NUPTs within the 3.19 Mbp male-specific Y region is congruent with findings in species such as *Asparagus officinalis*, where the male-specific region of the Y chromosome lacked NUPT insertions [83].

## Conclusions

We present high-quality, contiguous assemblies of the nuclear, chloroplast, and mitochondrial genomes of *A. tuberculatus*, including a chromosome-level nuclear assembly. These genomic resources provide a valuable foundation for exploring the evolution of the sex-associated region, diversity within the PEBP protein family, and nuclear DNA segments of organellar origin in this troublesome dioecious weed. Our analyses suggest that chromosome 1, which contains the sex-associated region, likely originated from the fusion of two ancestral chromosomes. While the absolute age of the sex-associated region is unknown, the ~ 0.5 MYA estimate for intact LTR insertions reflects subsequent TE accumulation dynamics rather than the origin itself. We further identified sex-linked and sex-biased differentially expressed genes with potential roles in sex determination and flowering. Together, these findings establish a framework for systematic comparative genomics across dioecious *Amaranthus* species to better understand the origin, variation, and evolution of sex-determining regions in the genus.

## Materials and methods

### Plant material and growth conditions

An accession of *A. tuberculatus*, designated WUS-BM and available through the USDA Germplasm Resources Information Network (GRIN: PI 698378), was used in this study. The original source population, designated the ABD population, from which WUS-BM was derived, was collected by Katherine E. Waselkov in 2009 at Aberdeen Community Park on the north bank of the Ohio River (GPS coordinates: 38.6543, −83.7623). Seeds from the accession were sown in presoaked potting soil (Lambert LM-GPS). Seedlings were grown to ~ 5 cm in height under a 16/8-h day/night photoperiod and 25/20 C temperature in the greenhouse prior to transplanting to 16-cm pots (America Clay Works I-A650MP). A single flowering male plant was placed in the dark for 72 h, after which 4 g and 2 g of fresh leaf tissues were sampled for PacBio HiFi and Hi-C library preparation, respectively. In each case, tissue was flash frozen in liquid nitrogen and stored at −80 C until use. Four grams of fresh leaf tissue was harvested from the same individual and shipped immediately on damp paper towels at 4 C for Bionano library preparation. Finally, a mix of four male and four female plants, assigned genotypically as described by [32], were grown as previously described. Root, stem, leaf, and meristem tissue were sampled from young (~ 6–10 leaf stage) and flowering plants and combined with floral tissue from several stages of development. All tissues were ground together in equal amounts by weight, and RNA was extracted using Zymo Direct-zol RNA Miniprep kit. Following quantification of concentration and quality, three µg of RNA was used for PacBio Iso-Seq library preparation. All samples were shipped on dry ice (except the tissue for Bionano library preparation) to the Genome Center of Excellence at Corteva Agriscience.

### Library preparation, genome sequencing, and assembly

DNA from a single *A. tuberculatus* individual was isolated from approximately 1.2 g of frozen leaf tissue using the Nucleobond HMW DNA kit (Macherey-Nagel GmbH & Co. KG, Düren, Germany) following the manufacturer’s protocol with some modifications [92]. Oxford Nanopore Technologies (ONT) ligation sequencing libraries were constructed using the LSK114 Kit (Oxford Nanopore Technologies, Oxford, UK) as per standard protocol with a few modifications. The libraries were loaded into FLO-PRO114M Flowcells, and data were collected using a PromethION 24 sequencer with Configuration v.5.8.6, MinKNOW v.23.11.7, Bream 7.8.2, and Dorado 7.2.13. In total, six flowcells were used to complete 12 runs yielding 2,305,539 reads with minimum length of 40 kbp, N50 of 54.7 kbp and total sequence length of 128,275,261,579 bp. Hi-C library was constructed using the Phase Genomics Proximo system following the manufacturer’s protocol (Phase Genomics, Seattle, WA). The library was then sequenced on an Illumina Novaseq 6000 system, yielding 142,208,874 PE150 paired clusters for a total sequence length of 43,862,662,200 bp. For Bionano optical mapping, nuclei were extracted from approximately 500 mg of young leaf tissue using Prep™ Plant Tissue DNA Isolation Kit (Bionano Genomics, San Diego, CA, USA). Ultra-high molecular weight (uHMW) DNA was then obtained from the resulting nuclei following resuspension and centrifugations (first at low speed of 100 x g, and subsequently at 2,500 x g). Bionano Direct Label and Stain (DLS) protocol was then carried out with 800 ng of the uHMW using a DLS kit. After the dye cleaning step, the DNA was loaded into a single Bionano chip in a Saphyr system following the manufacturer’s recommendations.

Filtered ONT simplex reads (104x) were assembled with hifiasm v0.21.0 with default parameters using the new module for ONT only assembly (--ont) [93]. The resulting primary contigs were filtered for minimum length (100 kbp) and minimum coverage (20x) thresholds yielding 64 filtered contigs (contig N50 = 34.6 kbp, sum = 1.20 Gbp). Contigs were combined with 70 Bionano maps (map N50 = 29.1 Mbp, sum = 1.20 Gbp) to create hybrid scaffolds using Bionano Access v1.8 and Bionano Solve v3.8, which were manually curated to a set of 32 scaffolds (scaffold N50 = 36.7 Mbp, sum = 1.195 Gbp). Hi-C data were aligned to scaffolds using Juicer v1.6 [94] to generate Hi-C contact maps. Visual inspection of the maps with Juicebox v1.11.08 [95] revealed that the 32 scaffolds represented 32 chromosomes. Homeologous scaffold/chromosome pairs were identified and oriented by chunking each scaffold into 100 bp “reads” and aligning to the other scaffolds using minimap2 v2.26 (using short read mode) [92]. The top matches were identified by visual inspection of the alignment coordinates with Tibco Spotfire (v14.0). Scaffolds were organized into 16 chromosome pairs, randomly designated as A (Hap1) and B (Hap2), with A representing the longer chromosome of each pair. Corrected ONT reads from hifiasm were aligned to the chromosome assembly using minimap2 (HiFi mode) to correct spurious consensus SNP/INDEL errors using samtools v1.3.1 [96]. In addition, manual curation was performed to resolve two overlapping contigs and one collapsed 327 kbp tandem repeat. The final assembly contains only 15 gaps, and the remaining gaps appear to be associated with AT-rich repeats and reduced ONT coverage.

### Genome annotation

The annotation pipeline had been previously described [97]. Briefly, transposable element families were first identified in the two chromosome-level assemblies using RepeatModeler v2.0.2 [98], RepeatMasker v4.1.2 (http://www.repeatmasker.org/RepeatMasker/), and BEDtools v2.30.0 [99]. Iso-Seq reads were then mapped to the softmasked assemblies generated from the repetitive elements’ annotation using pbmm2 v1.10.0. Redundant transcripts were collapsed using IsoSeq3 v3.8.2 (https://github.com/PacificBiosciences/IsoSeq). The collapsed gene models together with protein sequences from *A. hypochondriacus* [100] were used for the final gene model predictions in MakerP v1.0. Functional annotation and subcellular localization of proteins were carried out utilizing series of databases, including InterProScan5 [101], Uniref50 [102], and NCBI nonredundant protein database. Transfer RNA (tRNA) genes were predicted using tRNAscan-SE v2.0.12 [103] within Maker. Transcription factors were identified using PlantTFDB v5.0 (http://planttfdb.gao-lab.org/prediction.php) [104].

### Genome characteristics and repeat analysis

The assembly quality and completeness for both haplotypes were accessed using the Benchmarking Universal Single-Copy Orthologs (BUSCOs) v5.8.2 [105]. Genome characteristics were computed using “agat_sp_statistics.pl” from AGAT Toolkit v1.4.1 [106] and “bbstats.sh” from BBTools [107]. Species-specific repeats in the two haplotype assemblies were first identified using RepeatModeler v2.0.6 [98]. The RepBase database (RepeatMaskerEdition-20181026) [108] was then combined with Dfam3.8 database in RepeatMasker, and queried using the famdb.py utility in RepeatMasker to obtain ‘viridiplantae’ repeats library. LTR structural analysis was carried out separately using LTR_harvest from genometools v1.6.0 with parameters: -minlenltr 100 -maxlenltr 7000 -mintsd 4 -maxtsd 6 -motif TGCA -motifmis 1 -similar 85 -vic 10 -seed 20 -seqids yes [109], and LTR_FINDER_parallel with default parameters [110]. The output from both LTR_harvest and LTR_FINDER_parallel were combined and passed to LTR_retriever v3.0.1 [111] to generate a non-redundant LTR library for each haplotype.

The substitution rate of 2.81e-9 synonymous substitutions per site per year for the Chenopodioideae plastid *rbcL* gene was specified with -u parameter within LTR_retriever to estimate the insertion age of LTR elements. The three libraries (species-specific consensus libraries of repeats, viridiplantae repeats library, and non-redundant LTR libraries) were combined for each of the haplotypes, and clustered using CD-HIT-EST v4.8.1 [112] with parameters: -c 0.8 -G 0.8 -s 0.9 -aL 0.9 -aS 0.9 -M 0 -T 48. The non-redundant libraries were then used to annotate repeats in the haplotype assemblies using RepeatMasker v4.1.7 with parameters: -s -gff -lib -e rmblast (http://www.repeatmasker.org/RepeatMasker/). Annotated repeats were summarized with RepeatMasker’s “buildSummary.pl” script, and the distribution of LTRs was estimated using the perl script “LTR_sum.pl” within LTR_retriever.

*De novo* quality of intergenic and repetitive sequence space of both haplotypes was also accessed using LTR assembly index (LAI) from the LTR retriever pipeline [111, 113]. Putative centromeric repeats in both haplotypes were identified by running StainedGlass [114] with default parameters (window = 5000 and mm_f = 10000) to determine sequence identity within tandem repeat arrays. Telomeric repeats were identified by searching the telomere repeat motif (TTTAGGG)_n_ (where *n* = 4) against both haplotypes using BLASTN [115] with parameters: -task blastn-short -db haplotypes -query primers -outfmt 6 -out output.

### Genome-wide association analysis for sex in waterhemp populations

A total of 353 individuals (175 females and 178 males) derived from an artificially generated mapping population [116] and previously genotyped using RAD-seq [30] were used for GWA analysis. The single-end reads were demultiplexed and processed using Stacks v2.68 [117]. Each sample was then aligned to Hap1 of the *A. tuberculatus* genome assembly using BWA-MEM v0.7.18 [118]. Individuals were grouped by sex, and the “gstacks” command in Stacks was used to build loci. We ran the “populations” command employing a minimal filtering (--min-maf 0.05) and then assessed the level of missingness in the two data groups using VCFtools (--missing-indv) following a previously described approach [119]. Samples having more than 60% missing data were excluded from further analysis, and the “populations” command rerun on the streamlined samples using more filtering criteria (--min-maf 0.05, -p 2, -r 0.4). GWA analysis was carried out using complementary approaches with the set of variants obtained after the filtering step above. First, we obtained kinship matrix using emmax-kin, and used the matrix as covariates for EMMAX association, which utilizes a mixed-model association method (parameters: -d 10, -v). Second, we obtained principal components (PC) using PLINK v1.90b7 [120], and then used the first 20 PCs as covariates for the association analysis in BLINK-C [121]. Sex of the individuals were used as phenotypes in the analysis. Calculation of f-statistics (*F*_*ST*_) was performed using VCFtools v0.1.16 [122].

### Location of previous sex markers and genes in the assemblies

BLASTN [115] was used to search previously reported primer sets (WHMS, MU-976, MU-657.2, and MU-533) that amplified a male-specific region of *A. tuberculatus* [30, 32] against both haplotype assemblies. Additionally, a 200 bp *FT* sequence, previously reported as male-specific and conserved among three other dioecious amaranths related to waterhemp [34], was searched against both haplotype assemblies using BLASTN. Reciprocal best hit (RBH) searches between genes on the previously identified male-specific contigs and genes from the haplotype assemblies were also carried out with MMseqs2 [123]. Putative homologs of genes within the male-specific Y region elsewhere in the genome were identified with MMseqs2 using parameters --min-seq-id 0.7 -c 0.8 --cov-mode 1.

### Synteny and intragenomic analysis

Collinear gene blocks between haplotype 1 and 2 assemblies were identified using MCScan [124] from the JCVI utility libraries v1.3.5 with a C-score cutoff of 0.99. Blocks on chromosome 1 were visualized and plotted with JBrowse [125]. MCScanX [126] was also used to investigate collinear blocks of genes within the haplotypes, and the output collinearity files, after converting to “.anchors” format, were plotted with Circos [127]. Haplotype sequences were aligned with Minimap2 v2.28-r1209 [128], further processed with SyRI [129] to identify structural rearrangements, and visualized using D-Genies [130]. Syntenic orthologues among the haplotypes and six chromosome-level *Amaranthus* species (*A. hypochondriacus* [100], *A. cruentus* [131], *A. tricolor* [55], *A. palmeri*, *A. retroflexus*, and *A. hybridus* [132]) were evaluated and visualized using GENESPACE v1.2.3 [133].

### Sequence divergence and detection of positive selection

We followed previously described protocols to test for the evidence of adaptive evolution in 608 single-copy orthologous genes on chromosome 1 [134, 135]. Briefly, each orthogroup on chromosome 1 from the previous Orthofinder run containing the seven *Amaranthus* species were aligned separately using custom Perl scripts, “multiple_sequence_splitter.pl” and “align_orthologs.pl” from Jeffares et al., (2014). The codon alignments for the 608 orthologs were then used as input for CODEML program in PAML package v4.10.7 [136]. A series of models were adopted to test for adaptive evolution. We first compared the simplest or null model against a nested alternative site model (M0 vs. M1a) that allows neutral sites (ω = 1) to be evaluated. We then asked if adding a third class with ω >1 fits the data better than a model with only two classes, ω < 1 or ω = 1 (M1a vs M2a), as certain sites could be under positive selection. We further used the branch models to determine if specific foreground branches have different ω from background branches (M0 vs. two-ratio).

Using the branch-site model (MA_null_, ω = 1 vs. MA, ω >1), we determined if the previously defined foreground branches are more likely to contain sites under positive selection (see Fig. S14 of schematic representation of trees with defined foreground and background branches). For all hypotheses tested, we implemented the mutation-selection model (FMutSel) with observed codon frequencies used as estimates (CodonFreq = 7, estFreq = 0). The FMutSel model has been indicated to account for mutation bias and selection affecting codon usage, and is preferable over other models [135, 137]. We compared per-gene nested models using likelihood-ratio tests, and *p*-values were adjusted using the Benjamini–Hochberg method [138] to account for multiple comparisons.

### Expression profiling and gene ontology (GO) enrichment analysis

mRNA-sequencing data for three tissue types (mature flowers, shoot apical meristem, and floral meristems) from a previous study [56] were mapped to Hap1 of the *A. tuberculatus* genome using STAR v2.7.11b [139]. Two replicates from the mature flower category were excluded due to low mapping quality (25.05% and 26.86% uniquely mapped reads for replicates 2 and 4), consistent with their removal in the previous study. Gene counts were obtained with featureCounts v2.0.8 from the subread package [140], and the differential expression (DE) between sexes for each tissue type was analyzed using edgeR [141]. Genes with CPM < 1 in at least two samples were removed, and counts were normalized using the TMM method. A negative binomial generalized log-linear model was fitted to the normalized counts, and genes were considered differentially expressed using edgeR’s *glmTreat* function with FDR < 0.05 and FC >1.2. Heatmaps of these differentially expressed genes were generated with pheatmap after log_2_-transformed normalization of counts in DESeq2 [142]. Translated protein-coding sequences (CDS) of Hap1 were annotated with eggNOG-mapper v2.1.12 [143], and GO enrichment was assessed with topGO (nodeSize = 10) using Fisher’s exact test and the “elim” algorithm. GO terms were considered significantly enriched at *p* < 0.01.

### PEBP gene family diversity

Protein sequences of *A. tuberculatus* haplotype assemblies from this study, *A. hypochondriacus* [100], *A. cruentus* [131], *A. tricolor* [55], *A. palmeri*, *A. hybridus*, and *A. retroflexus* [132], and *Beta vulgaris* ssp. *vulgaris* [144] were retrieved for candidate PEBP classification. Two complementary approaches were utilized. First, protein sequences encoding the six PEBP genes in *A. thaliana* TAIR10 (Arabidopsis TAIR database) were searched against the *Amaranthus* species and *B. vulgaris* using BLASTP (e-value of 1e-5). Second, the Hidden Markov Model (HMM) for PEBP family (PF01161) retrieved from Pfam (http://pfam.xfam.org, accessed on January 29, 2025) were searched against *Amaranthus* species and *B. vulgaris* protein databases using hmmsearch from HMMER v3.4 [145]. All hits from the HMM search were further verified for the conserved PEBP domain using CDD (https://www.ncbi.nlm.nih.gov/Structure/bwrpsb/bwrpsb.cgi). Predicted protein sequences lacking a PEBP domain were excluded from further analysis. Additionally, the twenty-three PEBP protein sequences previously reported for *Chenopodium quinoa* [75, 77] and PEBP sequences of *Spinacia oleracea* retrieved from Phytozome v14 [146, 147] were included for alignment. PEBP protein sequences from all species were then aligned using MAFFT v7.511 [148]. Phylogenetic tree of the PEBP proteins was constructed using the maximum-likelihood method in RAxML v8.2.13 under the JTT + GAMMA model, selected via PROTGAMMAAUTO, with 1000 rapid bootstrap replicates. The phylogenetic tree was midpoint-rooted, visualized, and annotated in iTOL v7 [149]. The gene structure of *FLOWERING LOCUS T* and its homologs were visualized using GSDS 2.0 [150].

### Organellar genomes and their homologous sequences in the nuclear genome

The chloroplast and mitochondrial genomes of *A. tuberculatus* were contiguously assembled as part of the assembly pipeline described previously for the nuclear genome. We, however, focused on the annotation of the mitochondrial genome of *A. tuberculatus* due to previous work on the chloroplast genome of the species [57, 58]. The assembled mitogenome was annotated with GeSeq [151] using BLAT, tRNAscan-SE v2.0.7, and NCBI RefSeq sequences from *B. maritima*, *B. vulgaris*, *S. oleracea* and *C. quinoa*. The BLAT search parameters for protein search identity and rRNA, tRNA, DNA search identity was set to 45% and 85%, respectively. The mitochondrial genome and the annotation were then visualized using the program OGDRAW [152]. Simple sequence repeats were identified using MISA v2.1 (https://webblast.ipk-gatersleben.de/misa/) with parameters: 12, 6, 4, 3, 3, and 3 for mono-, di-, tri-, tetra-, penta-, and hexanucleotide repeats, respectively [153]. Repetitive sequences were further identified with REPuter (https://bibiserv.cebitec.uni-bielefeld.de/reputer) with parameters: 30 bp (minimum repeat size), 100 (maximum computed repeats), 3 (hamming distance) [154]. Previously reported mitochondrial genomes of *C. quinoa* (GenBank accession MK182703.1), *S. oleracea* (GenBank accession NC_035618.1) and *B. vulgaris* ssp. *vulgaris* (GenBank accession NC_002511.2) were obtained from NCBI and compared to the *A. tuberculatus* mitochondrial genome using minimap2 [92]. Alignments were visualized as dotplots with D-GENIES [130].

To identify nuclear sequences of organellar origin and fragments transferred between organelles (i.e., from chloroplast to mitochondrion), two complementary approaches were utilized. First, coding sequences (CDS) from the mitogenome and plastome were searched against the nuclear genome, and the coding sequences from the plastome were searched against the mitogenome using BLASTN [115]. Second, the complete mitogenome and plastome were searched against the nuclear genome using BLASTN, allowing for the identification of longer homologous blocks or fragments inserted into the nuclear genome. The BLAST parameters used included a minimum identity threshold of 80%, e-value cutoff of 1 × 10^−5^, and alignment lengths exceeding 100 bp. The BLAST outputs were manually filtered to remove overlapping or redundant matches.

### Statistical analysis

Gene count per 500 kbp, TE proportion per 500 kbp, and LTR insertion times data were analyzed using Kruskal-Wallis test. Post-hoc test of multiple comparisons between regions (Collinear 1, INS 1, INV 1, Collinear 2, INV 2, Collinear 3) was carried out with Conover-Iman test, following the rejection of the null hypothesis from the Kruskal-Wallis test. *P*-values were adjusted for multiple comparisons with Benjamini–Hochberg correction [138]. The statistical analyses were carried out with the PMCMRplus package in R v4.1.2 [155].

## Supplementary Information

Supplementary Material 1.

Supplementary Material 2.

## Data Availability

Sequencing data and the genome assemblies in this study are available through the National Center for Biotechnology Information (NCBI) under BioProject PRJNA1089550. The genome assemblies and annotations are also available on CoGe under accession IDs 69059 and 69062. The mitochondrial and chloroplast genomes assemblies in this study were submitted along with the nuclear genomes under BioProject PRJNA1085752. PEBP protein sequences, MAFFT alignment, and RAxML tree are available on figshare 10.6084/m9.figshare.29856191.

## Abbreviations

BLINK: Bayesian-information and linkage disequilibrium iteratively nested keyway
CDD: Conserved domain database
EMMAX: Efficient mixed-model association eXpedited
LTR-RTs: Long terminal repeat retrotransposons
MSY: Male-specific Y region
NUMTs: Nuclear mitochondrial DNA segments
NUPTs: Nuclear plastid DNA segments
PAR: Pseudoautosomal region
PEBP: Phosphatidylethanolamine binding protein
RAD-GWA: Restriction site-associated DNA Genome-wide association
SDR: Sex-determining region
TMM: Trimmed Mean of M-values
