# Supplementary material for: Analyses of contiguous reference genomes of Amaranthus tuberculatus highlight the landscape of the sex-associated region and PEBP gene family diversity
Source: BMC Genomics. 2025 Nov 3;26:988. doi: 10.1186/s12864-025-12181-w (PMC12581244; doi:10.1186/s12864-025-12181-w)
Supplement: Supplementary file 2 — Supplementary Material 2. [file 12864_2025_12181_MOESM2_ESM.docx]

**Supporting Information**

Article title: Analyses of contiguous reference genomes of *Amaranthus tuberculatus* highlights the landscape of sex-determining region, PEBP gene family diversity, and organelle-derived nuclear insertions

Authors: Damilola A. Raiyemo^1^, Luan Cutti^2^, Eric Patterson^2^, Victor Llaca^3^, Kevin Fengler^3^, Jacob S. Montgomery^4^, Sarah Morran^4^, Todd A. Gaines^4^ and Patrick J. Tranel^1,*^

*Correspondence: [tranel@illinois.edu](mailto:tranel@illinois.edu)

The following Supporting Information is available for this article:

**Fig. S1** Heatmaps of tandem repeat structures for each of the 16 chromosome of haplome 1 assembly. Higher-order repeats represented by brightly-colored region indicates the centromere.

**Fig. S2** Heatmaps of tandem repeat structures for each of the 16 chromosome of haplome 2 assembly. Higher-order repeats represented by brightly-colored region indicates the centromere.

**Fig. S3** Search of the 572 bp male-specific marker to a database of transposable element encoded proteins. The marker matched to two hits with about 79.54% or 94.23% of the sequences masked. Softmasked sequences are represented with lowercase.

**Fig. S4** Genomic features of the two haplotype assemblies of *A. tuberculatus*. (a) Dotplot of base alignment between the two haplomes. (b) synteny pattern between both haplomes indicating a 1:1 relationship in gene content.

**Fig. S5** Gene structure of *FT* and its homologs in Hap1 of the *A. tuberculatus* genome assembly showing exon-intron organization.

**Fig. S6** Schematic representation of trees displaying branches used as foreground (red color) in CODEML analysis. (a) Haplotype 1 was used as the foreground branch while others were background branches. (b) Haplotype 2 was used as the foreground branch while others were the background branches. (c) Both haplotypes were used as the foreground branches (d) Both haplotypes including the branch leading to their common ancestor were used as foreground branches.


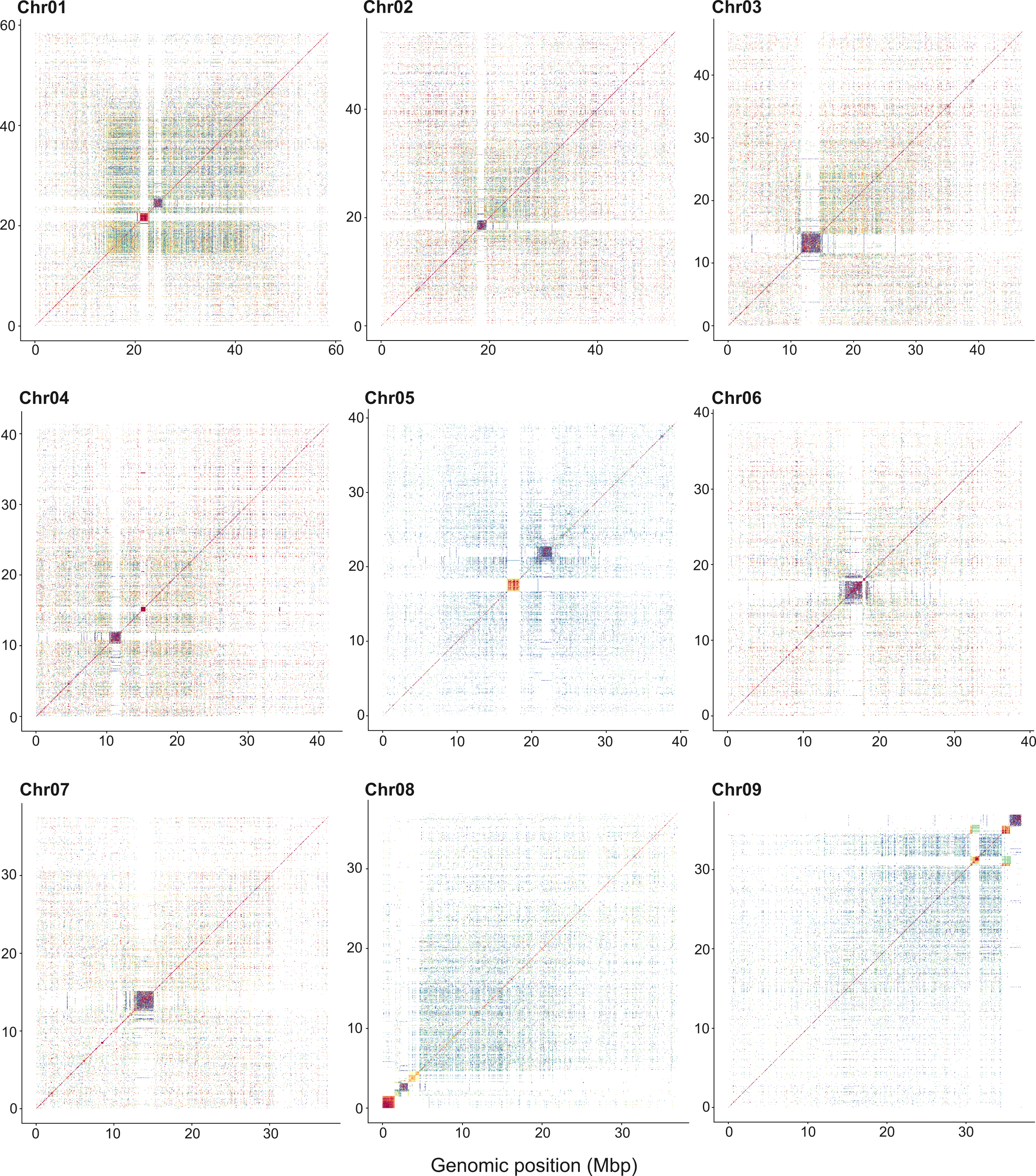


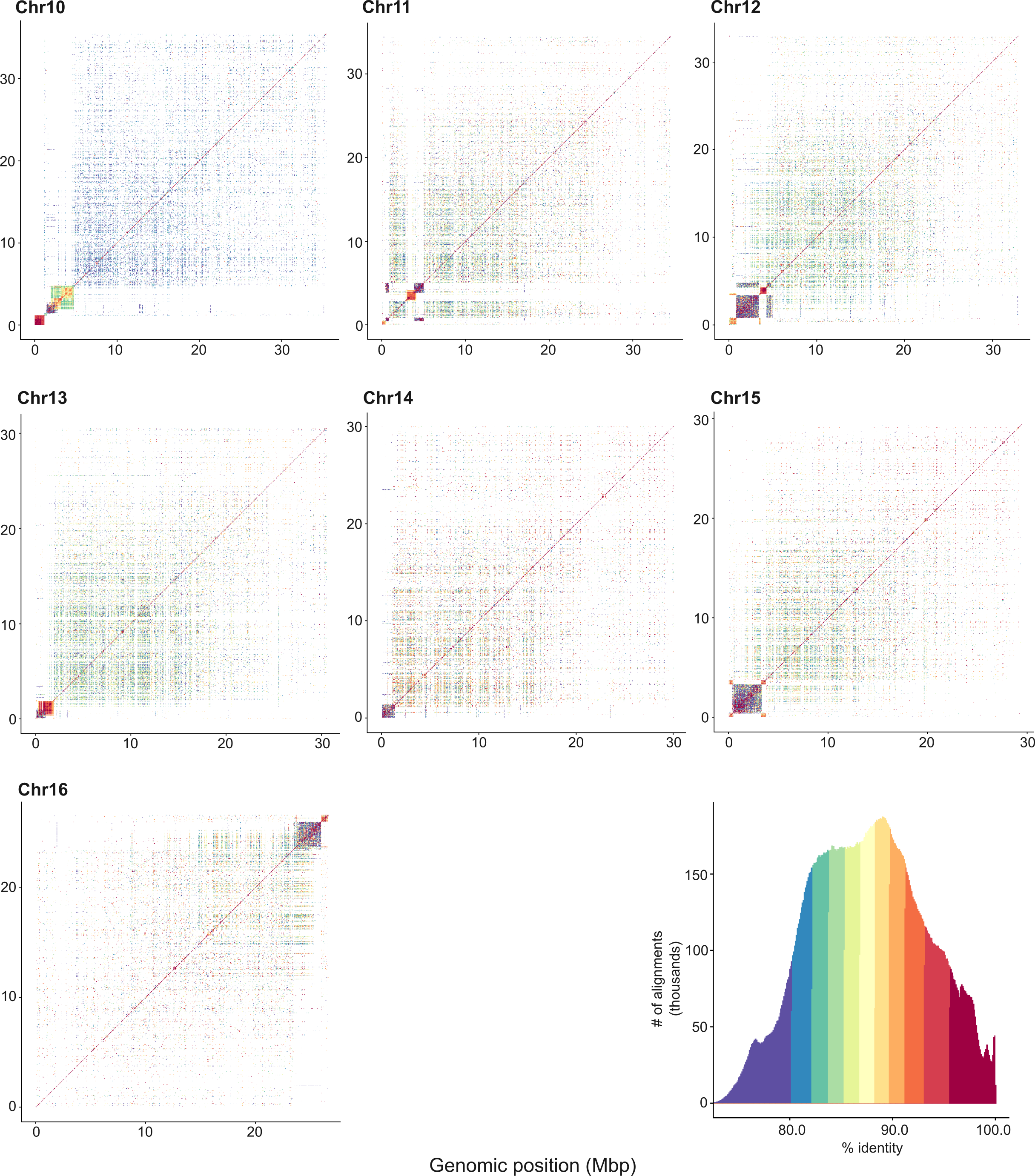


**Fig. S1** Heatmaps of tandem repeat structures for each of the 16 chromosome of haplome 1 assembly. Higher-order repeats represented by brightly-colored region indicates the centromere.


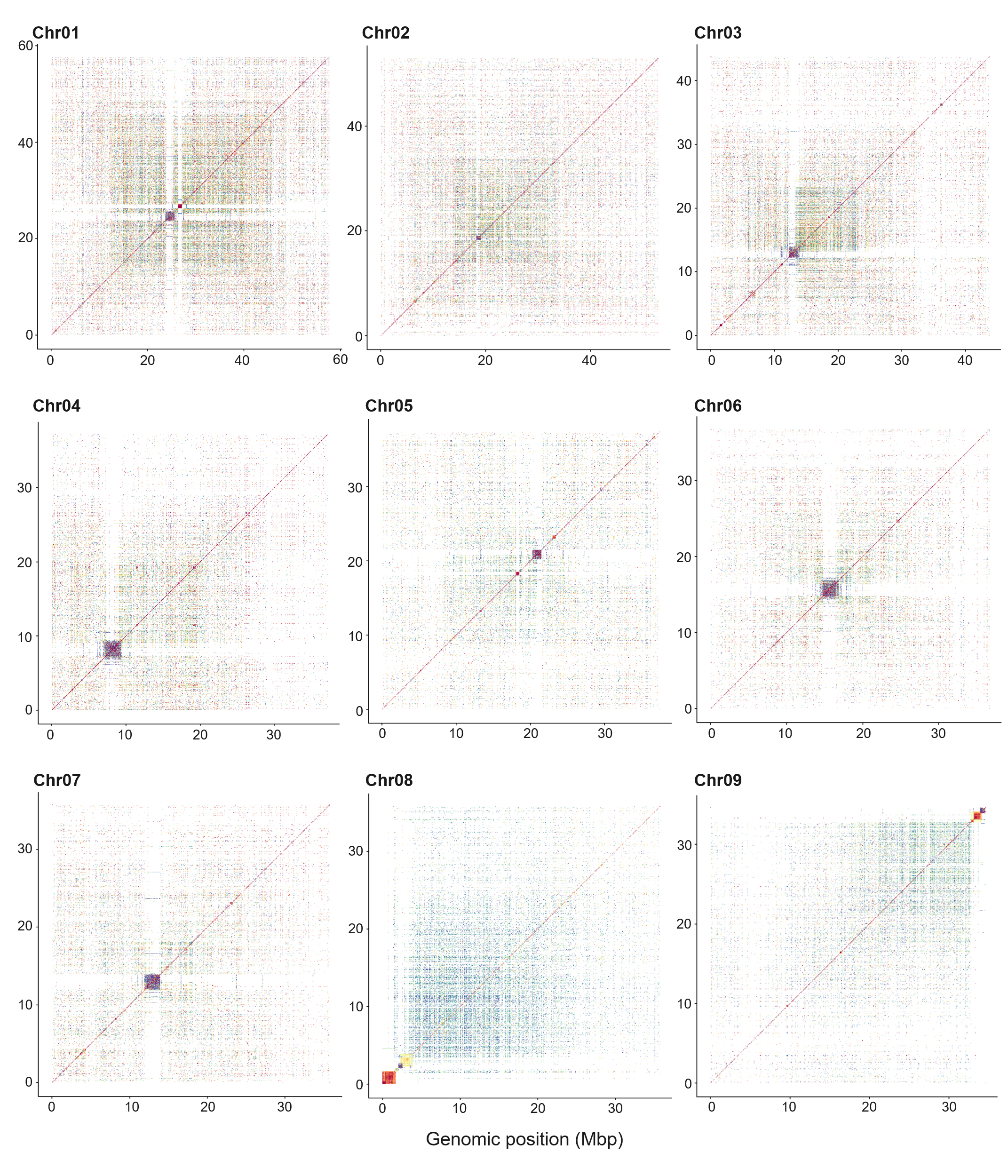


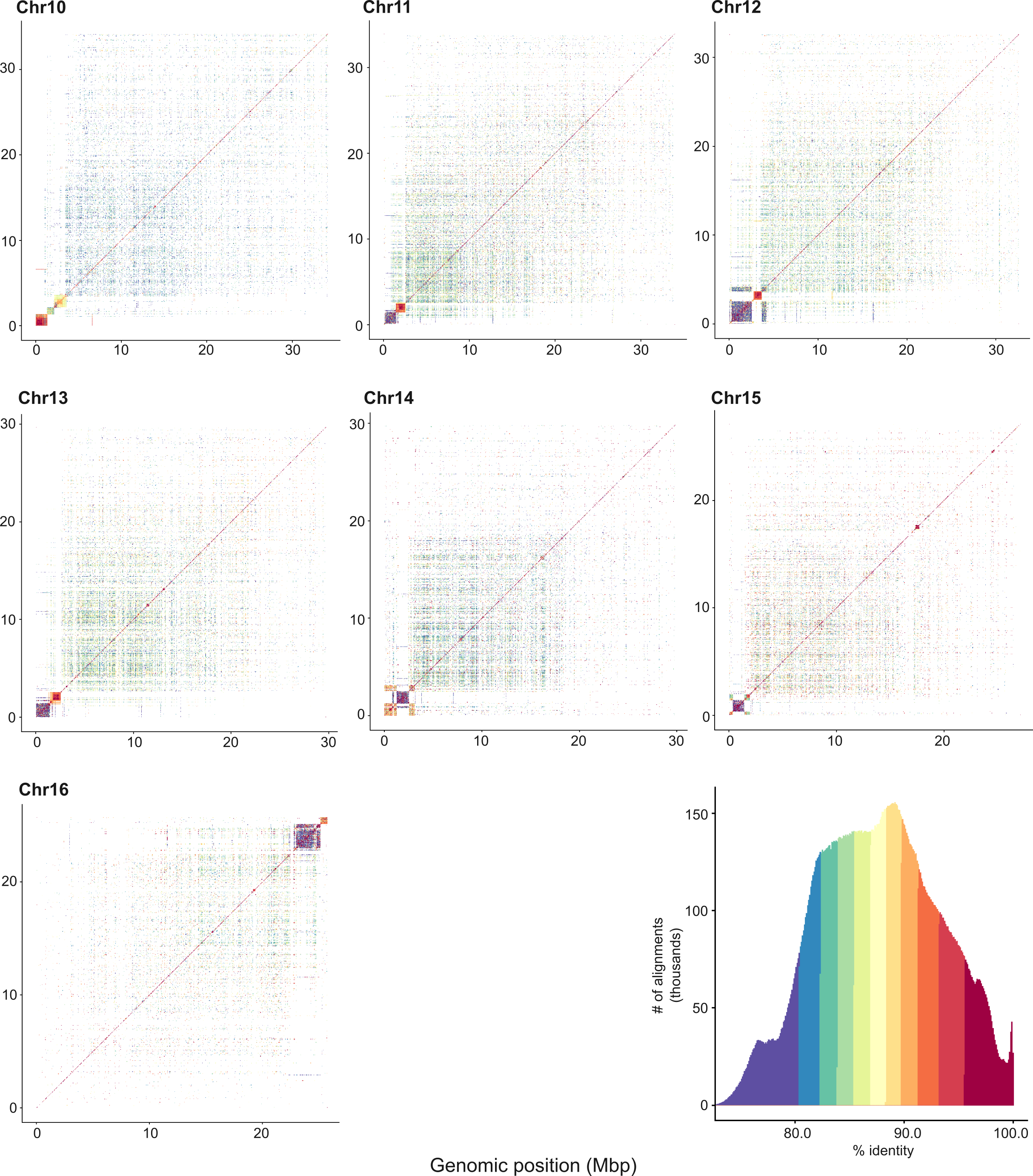


**Fig. S2** Heatmaps of tandem repeat structures for each of the 16 chromosome of haplome 2 assembly. Higher-order repeats represented by brightly-colored region indicates the centromere.


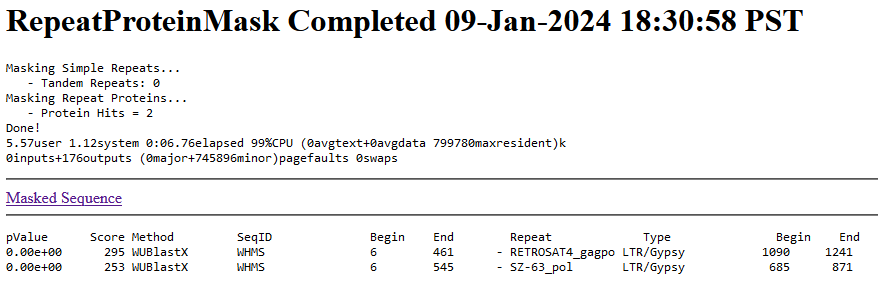


>WHMS_572bp_marker

atgacatcattccctcactgagtcctaagtagtcaaaccacaaaatccatagagatcgaa

tcccacttccaatcagggacaagcaagggttgaagcagtcctgaactctttttccttttg

aattttacctgctgacataataaacacttagacacaaactctgatacatcttttcttagc

cctttccaccagattagctctctaatttcttcaatcattttatcacgaccaggatgtaaa

tgaaacaagctttgagtacctactttcaaaatgtcattcttcaactcgagttgatttgga

acgcacAACCTACCATTCATCCTTAGCTCCCCTCTCTCCCCCAACTCAAAAGCATTAATc

ttattagcttcatctctatgtatcaattccacaacctctagatcctcaagttgtgcctca

atgatatgatcaaacaaggacagttgcacagtcattgctcccaaggagtgaccaacctct

ggccgtgtaaaatctctaacccaaagttctgcacctcacgatacaactccCATGGTACAG

TCATCAAAGCATTTAGACTcactcttggtctcc

**Fig. S3** Search of the 572 bp male-specific marker to a database of transposable element encoded proteins. The marker matched to two hits with about 79.54% or 94.23% of the sequences masked. Softmasked sequences are represented with lowercase.


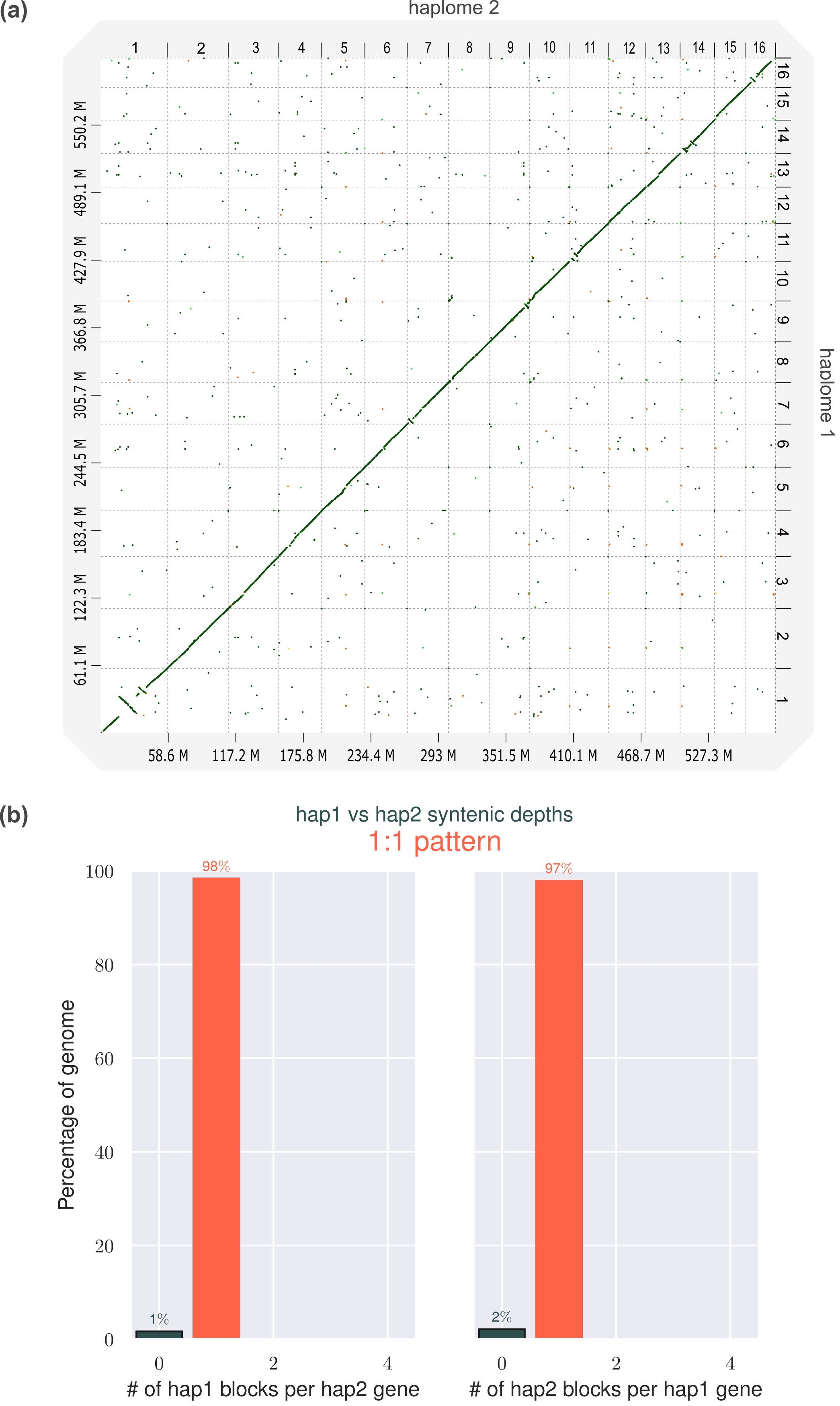


**Fig. S4** Genomic features of the two haplotype assemblies of *A. tuberculatus*. (a) Dotplot of base alignment between the two haplomes. (b) synteny pattern between both haplomes indicating a 1:1 relationship in gene content.


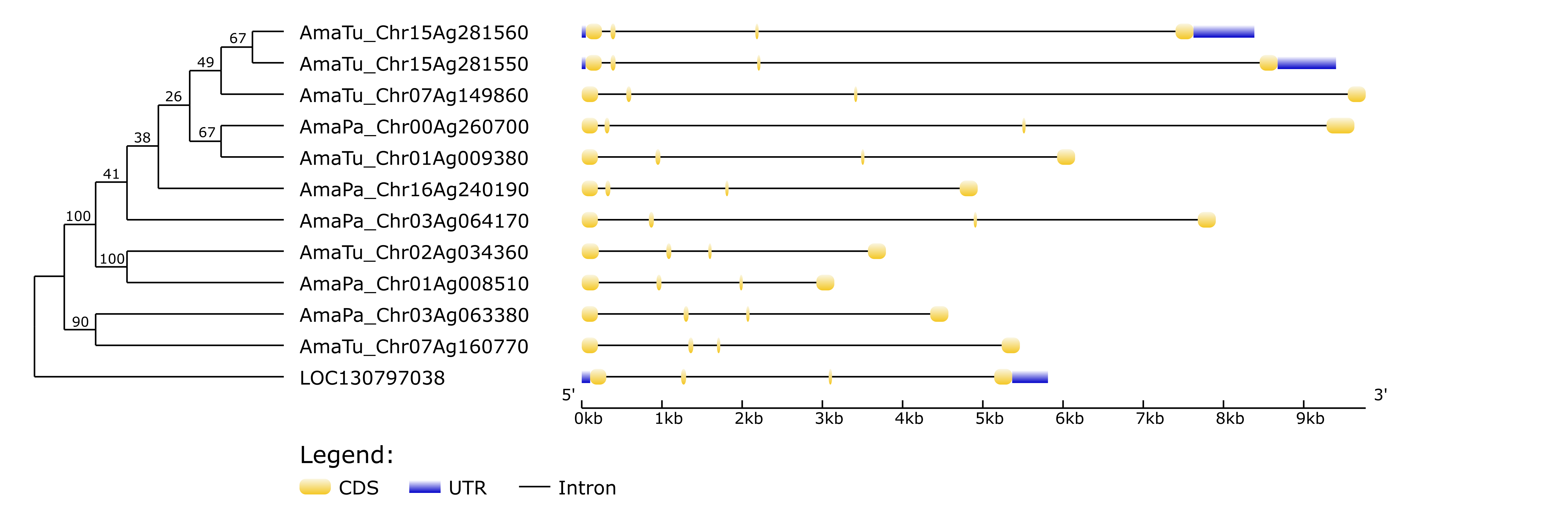
**Fig. S5** Phylogenetic tree of FT protein sequences and gene structures in Hap1 of *A. tuberculatus* and *A. palmeri* genome assembly showing exon-intron organization. The tree was rooted with an FT homolog from *A. tricolor*. Values on the tree represent RAxML bootstarp support (BS) values.

**
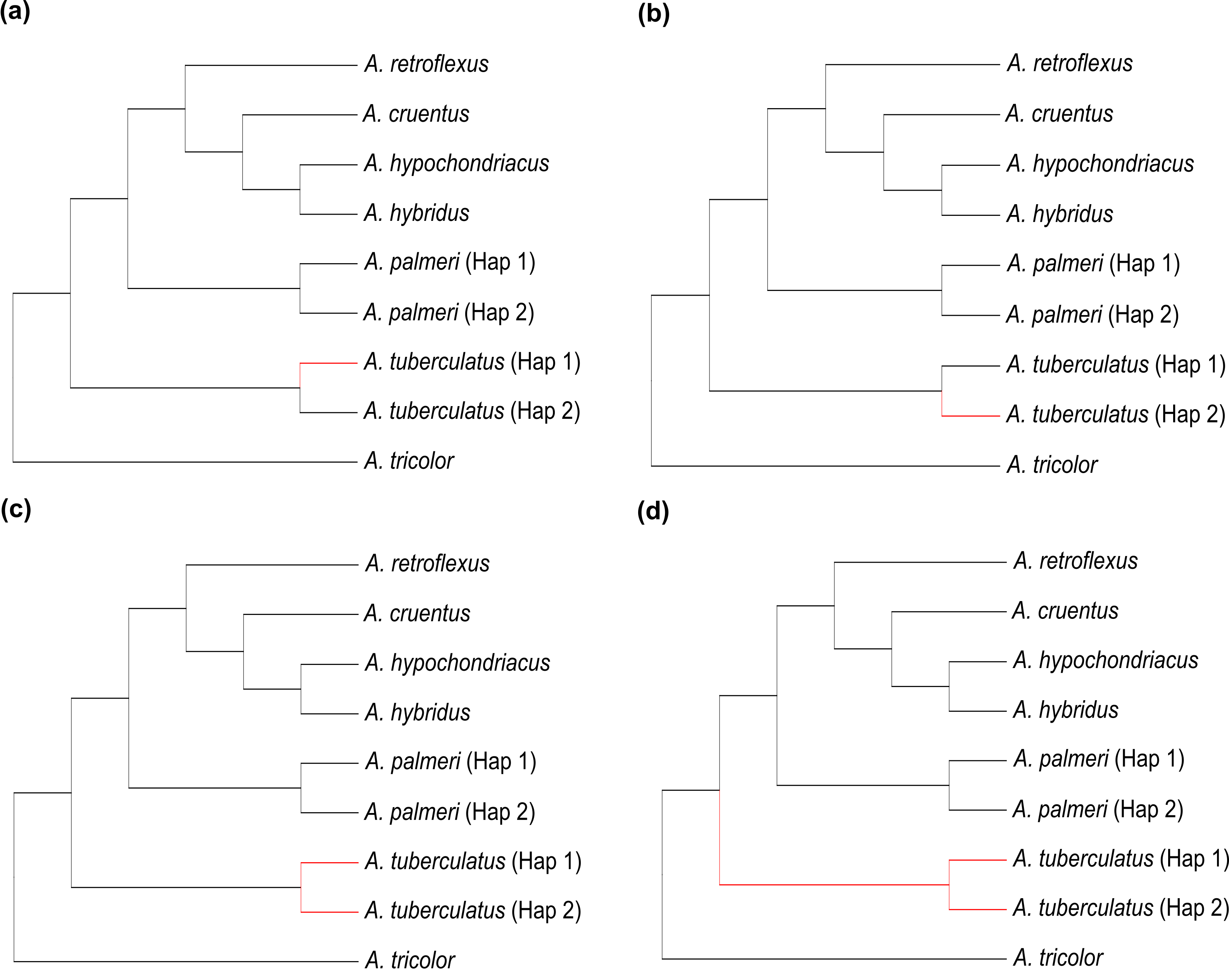
**

**Fig. S6** Schematic representation of trees displaying branches used as foreground (red color) in CODEML analysis. (a) Haplotype 1 was used as the foreground branch while others were background branches. (b) Haplotype 2 was used as the foreground branch while others were the background branches. (c) Both haplotypes were used as the foreground branches (d) Both haplotypes including the branch leading to their common ancestor were used as foreground branches.
